# Transcriptomic and metabolite analyses of Cabernet Sauvignon grape berry development

**DOI:** 10.1186/1471-2164-8-429

**Published:** 2007-11-22

**Authors:** Laurent G Deluc, Jérôme Grimplet, Matthew D Wheatley, Richard L Tillett, David R Quilici, Craig Osborne, David A Schooley, Karen A Schlauch, John C Cushman, Grant R Cramer

**Affiliations:** 1 Department of Biochemistry and Molecular Biology, University of Nevada, Reno, Nevada 89557-0014, USA; 2 Department of Animal Biotechnology, University of Nevada, Reno, NV 89557-0014, USA; 3 Boston University School of Medicine, Department of Genetics and Genomics, Boston University, E632, Boston, MA 02118, USA

## Abstract

**Background:**

Grape berry development is a dynamic process that involves a complex series of molecular genetic and biochemical changes divided into three major phases. During initial berry growth (Phase I), berry size increases along a sigmoidal growth curve due to cell division and subsequent cell expansion, and organic acids (mainly malate and tartrate), tannins, and hydroxycinnamates accumulate to peak levels. The second major phase (Phase II) is defined as a lag phase in which cell expansion ceases and sugars begin to accumulate. Véraison (the onset of ripening) marks the beginning of the third major phase (Phase III) in which berries undergo a second period of sigmoidal growth due to additional mesocarp cell expansion, accumulation of anthocyanin pigments for berry color, accumulation of volatile compounds for aroma, softening, peak accumulation of sugars (mainly glucose and fructose), and a decline in organic acid accumulation. In order to understand the transcriptional network responsible for controlling berry development, mRNA expression profiling was conducted on berries of *V. vinifera *Cabernet Sauvignon using the Affymetrix GeneChip^® ^*Vitis *oligonucleotide microarray ver. 1.0 spanning seven stages of berry development from small pea size berries (E-L stages 31 to 33 as defined by the modified E-L system), through véraison (E-L stages 34 and 35), to mature berries (E-L stages 36 and 38). Selected metabolites were profiled in parallel with mRNA expression profiling to understand the effect of transcriptional regulatory processes on specific metabolite production that ultimately influence the organoleptic properties of wine.

**Results:**

Over the course of berry development whole fruit tissues were found to express an average of 74.5% of probes represented on the *Vitis *microarray, which has 14,470 Unigenes. Approximately 60% of the expressed transcripts were differentially expressed between at least two out of the seven stages of berry development (28% of transcripts, 4,151 Unigenes, had pronounced (≥2 fold) differences in mRNA expression) illustrating the dynamic nature of the developmental process. The subset of 4,151 Unigenes was split into twenty well-correlated expression profiles. Expression profile patterns included those with declining or increasing mRNA expression over the course of berry development as well as transient peak or trough patterns across various developmental stages as defined by the modified E-L system. These detailed surveys revealed the expression patterns for genes that play key functional roles in phytohormone biosynthesis and response, calcium sequestration, transport and signaling, cell wall metabolism mediating expansion, ripening, and softening, flavonoid metabolism and transport, organic and amino acid metabolism, hexose sugar and triose phosphate metabolism and transport, starch metabolism, photosynthesis, circadian cycles and pathogen resistance. In particular, mRNA expression patterns of transcription factors, abscisic acid (ABA) biosynthesis, and calcium signaling genes identified candidate factors likely to participate in the progression of key developmental events such as véraison and potential candidate genes associated with such processes as auxin partitioning within berry cells, aroma compound production, and pathway regulation and sequestration of flavonoid compounds. Finally, analysis of sugar metabolism gene expression patterns indicated the existence of an alternative pathway for glucose and triose phosphate production that is invoked from véraison to mature berries.

**Conclusion:**

These results reveal the first high-resolution picture of the transcriptome dynamics that occur during seven stages of grape berry development. This work also establishes an extensive catalog of gene expression patterns for future investigations aimed at the dissection of the transcriptional regulatory hierarchies that govern berry development in a widely grown cultivar of wine grape. More importantly, this analysis identified a set of previously unknown genes potentially involved in critical steps associated with fruit development that can now be subjected to functional testing.

## Background

Grapes have been cultivated and fermented into wine for more than 7,000 years. Worldwide, grapes are one of the most widely cultivated fruit crops, encompassing 7.4 million hectares of arable land in 2006 [1] and with 68.9 million metric tons produced in 2006, ranks second among bananas, oranges, and apples with 69.7, 63.8 and 62.1 million metric tons respectively, produced during this same period. However, because the majority of the grapes that are harvested are fermented into wine, the economic impact for this commodity is far greater than the value of the grapes. For example, wine sales from California alone in 2006 was at an all-time high and growing with approximately $18 billion dollar in sales [2]. According to 2005 statistics, the California wine industry has a $52 and $125 billion economic impact on the state and U.S. economies, respectively [3].

In addition to their economic importance, consumption of grapes and wine has numerous nutritional and health benefits for humans [4,5]. For example, there are more than 200 polyphenolic compounds in red wines that are thought to act as antioxidants. In particular, one antioxidant compound, trans-resveratrol, has been shown to play a role in the prevention of heart disease (atherosclerosis) [6] and cancer [7]. Resveratrol slows the aging process in animals [8], acts as a signaling molecule in the brain [9], and down-regulates the expression of genes that are involved in cell cycle and cell proliferation in human prostate cells [10]. Therefore, for a variety of reasons, there is great interest in manipulating grape berry development and quality for both economic and health reasons.

In contrast to the well studied climacteric fruits such as tomato and apple, very little is known about the development and ripening processes of non-climacteric fruits such as grape or strawberry [11,12]. In 1992, Coombe, one of the leaders in the field, described our knowledge of grape berry development and the regulation of ripening as "embryonic [13]."

Grape berries, like other berry fruits, undergo a complex series of physical and biochemical changes during development, which can be divided into three major phases [13] with more detailed descriptive designations, known as the modified E-L system, being used to define more precise growth stages over the entire grapevine lifecycle [14]. During the initial stage of berry growth (Phase I) cell division is rapid and all cells are established in the developing fruit in the first two weeks after flowering followed by a subsequent sigmoidal increase in berry size over approximately 60 days due to cell expansion. Two important organic acids, tartrate and malate, are synthesized and reach maximal concentrations by the end of Phase I. Biosynthesis of tannins and hydroxycinnamates, which are major precursors for phenolic volatiles, also occurs, primarily during Phase I. Tannins are located primarily in the skin and seeds of the berry, and are perceived as astringent compounds important for color stability and the body of red wine.

Phase II is characterized as a lag phase during which there is no increase in berry size. Biosynthetic processes are not well characterized for this stage, but it is known that sugar accumulation begins during this phase just prior to véraison (the onset of ripening) [13]. Véraison marks the start of Phase III of berry growth, which is characterized by the initiation of color development (anthocyanin accumulation in red grapes) and berry softening. Berry growth is sigmoidal during Phase III, as the berries double in size. At the onset of this stage, sugars (largely glucose and fructose) continue to accumulate, and organic acid concentrations decline. The acid:sugar balance at harvest is critical for high quality wines, as it affects important sensory attributes [15]. A large number of the flavor compounds and volatile aromas are synthesized at the end of Stage III. Many of these aromas are derived from terpenoids. However, the availability of seed tannins declines through oxidative processes during Phase III, causing the tannins to bind to the seed coat, reducing the astringent components within the berry. Skin tannins begin to interact and bind with anthocyanins and each other, increasing tannin polymer size and complexity.

Two major objectives of modern viticultural practices include the ability to produce a uniformly ripe crop and to harvest at optimal grape maturity. Large variations in ripening among berries within a cluster and within a vineyard make it difficult to determine when a crop is at its best possible ripeness. The start of véraison is recognized to be a critical determinant for berry harvest dates, yet little is known about what initiates this important stage. A more detailed understanding of the complex changes in gene expression that orchestrate berry developmental processes is needed.

Several mRNA expression-profiling studies have been completed for *Vitis *berries. Differential screening of cDNA libraries from (*Vitis vinifera *cv. Shiraz) and northern blot analysis revealed that large differences in gene expression occur during berry ripening and led to the isolation of a large number of grape ripening-induced protein (GRIP) genes [16]. Monitoring of gene expression profiles in flowers and across six time points during grape (*Vitis vinifera *cv. Shiraz) berry skin development to 13 weeks post-flowering resolved four sets of genes with distinctive and similar expression patterns using spotted cDNA microarrays containing 4,608 elements [17]. mRNA expression was also studied across nine stages of wildtype cv. Shiraz berry development (green "pea" to overripe) [18] and in a fleshless berry mutant cv. Ugni Blanc using oligonucleotide microarrays containing 3,200 elements [19]. Differences in transcript expression profiles in the skin of ripening fruit (12 to 13 weeks after flowering) of seven different cultivars were also examined using a 9,200 feature cDNA microarray [20]. In this study, we conducted mRNA expression profiling on one of the widely grown varieties of *V. vinifera *(cv Cabernet Sauvignon) using the *Vitis *Affymetrix GeneChip^® ^oligonucleotide microarray ver. 1.0, which contains 14,470 Unigenes, over seven temporal stages (green "pea" to ripe) of berry development. We also correlated specific transcript profiles with specific metabolite profiles to gain deeper insights into discrete aspects of grape berry developmental dynamics.

## Results and discussion

### Grape berry development

**Figure 1:**
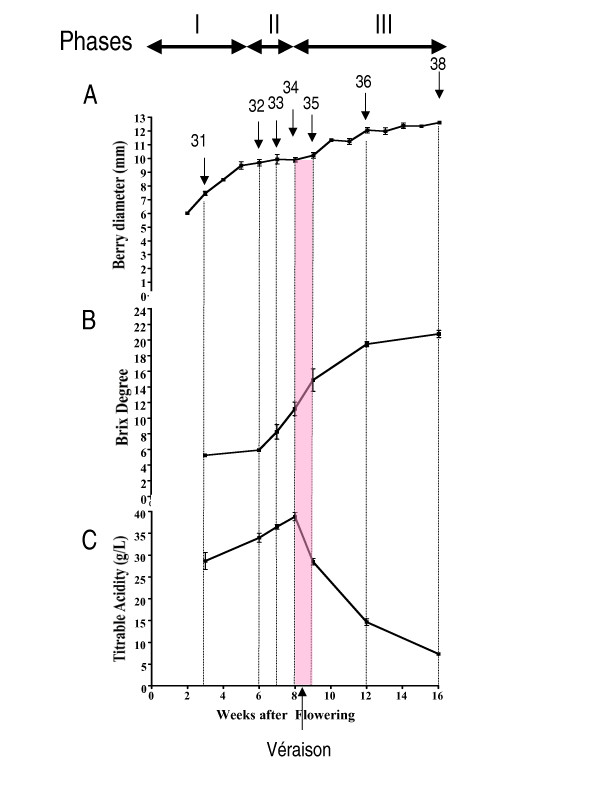
**Physiological data at different stages of berry development**. Changes in physiological parameters measured during the major phases (I to III) of berry development and ripening of Cabernet Sauvignon grape berries. A, Berry Diameter (*n *= 6); B, Brix degree (°) or total soluble solids in the berry juice (*n *= 6); C, Titratable Acidity (g/L) (*n *= 6). The stage at which véraison occurs is indicated in pink. Numbers with arrows point to the individual developmental stages defined by the E-L system Coombe [14] used for transcriptome profiling.

*Vitis vinifera *cv. Cabernet Sauvignon grapes were harvested on a weekly basis over the course of berry development from the Shenandoah Vineyard, Plymouth, California during the summer of 2004. Samples corresponding to stages 31 to 38 of the modified E-L system [14] were measured for berry diameter, °Brix (an approximate measure of the mass ratio of dissolved solids, mostly sucrose, to water in fruit juices) and titratable acidity (Figure 1). Berry diameter increased over time with a classical double sigmoid pattern (Figure 1A). Average berry diameter increased during the first 7 weeks of development (E-L stage 31), followed by a cessation of berry expansion at 7 to 8 weeks post-anthesis (E-L stages 32 to 34), and then the increase in berry diameter resumed until maturity (E-L stages 35 to 38). °Brix increased 6 weeks post-anthesis to a peak value of 22 °Brix at 16 weeks post-anthesis (Figure 1B). In contrast, titratable acidity (g/L), which reflects acid accumulation (mainly tartaric and malic acid), increased steadily up to 8 weeks post-anthesis and then sharply declined at the start of véraison between E-L stages 34 and 35 reaching approximately 7 g/L of titratable acids at harvest (Figure 1C).

### Microarray analysis

The mRNA expression profiles of seven time points spanning E-L stages 31 to 38 as indicated in Figure 1 were compared using the Affymetrix GeneChip^® ^*Vitis *genome array ver. 1.0. Testing was performed using biological triplicates for each time point. Multiple time points within Stage II (E-L stages 32 to 35) were sampled due to the large number of biochemical changes expected to occur around véraison that affect berry ripening and fruit quality. A visual inspection of the distributions of raw perfect match (PM) probe-level intensities for all 21 arrays showed that the pre-processed and normalized PM intensities using Robust Multi-Array Average (RMA) [21] were consistent across all arrays. Digestion curves describing trends in RNA degradation between the 5' end and the 3' end in each probe set were examined and all 21 proved very similar [Additional File 1A,B]. Correlations among biological replicates were good: Spearman coefficients ranged from 0.977 to 0.997; Pearson coefficients ranged between 0.977 and 0.996.

From the *Vitis *16,602 probesets represented on the array [Additional File 1C], an overall mean call rate of 74.5% per array (range 73.5% to 76.2%) was obtained. Data from the 12,596 probe sets that were found to be present in at least two out of the three biological triplicates were retained for further analyses. After performing an ANOVA and a multiple testing correction (Benjamini and Hochberg) [22], we found that 10,068 probesets (60.6%) were differentially expressed (p ≤ 0.05) between two or more E-L stages of berry development [Additional File 2: Table 1]. Because one Unigene can be related to several probesets, the number of Unigenes decreased to 9,143 Unigenes [Additional File 2: Table 2]. These probesets will be hereby referred to as those passing the ANOVA filter. From this set of genes, we extracted a subset of 4,510 probesets that displayed a two-fold or greater change in steady-state transcript abundance over the course of development (i.e., across any two of the seven developmental stages) [Additional File 2: Table 3] representing 4,151 Unigenes (28.3%) in the DFCI Grape Gene Index database VvGI5 [23]. We refer to this subset of genes as the two-fold ratio (TFR) set [Additional File 2: Table 4].

Principal component analysis (PCA), was used to simplify and define associations between different developmental stages within the global transcriptomic data (Additional File 3). Two principal components explaining 97.4% of the overall variance of transcription profiles (86.8% and 7.6% for axes 1 and 2, respectively) allowed us to clearly differentiate E-L stages 31 and 35 from the other developmental stages analyzed (Additional File 3). It was not possible to clearly separate E-L stages 32 to 34 or 36 to 38 indicating that the transcription patterns occurring at these stages were similar to one another. However, stage 35, which corresponds to early post-véraison, could be distinguished suggesting that transcription patterns at this point in berry ripening are unique to this critical stage in berry development. Further analysis using a third axis explaining 2.7% of the overall variance, confirmed the previous results and slightly improved the resolution among stages 31, 35, and 36 to 38.

### Clustering of significant genes

**Figure 2:**
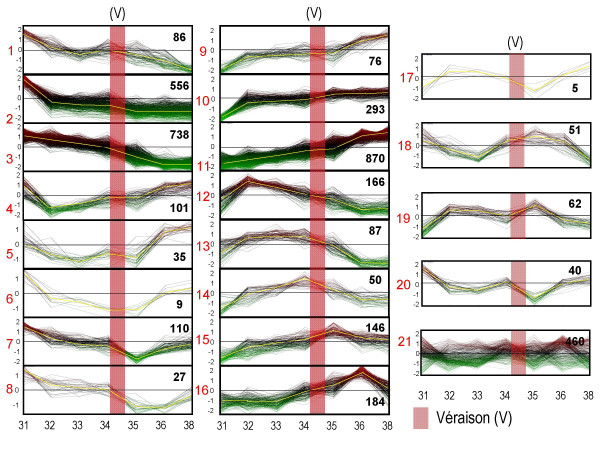
**Twenty-one profiles of steady-state transcripts exhibiting a two-fold or greater expression across berry development**. Profiles are plotted as RMA data values plotted on the log_2 _scale centered by the mean of all values (Stage 31 to stage 38). E-L Stages are indicated along the X-axis. Profiles numbers are indicated with red numbers with the number of transcripts within each profile indicated with black numbers: Véraison (V) is indicated with a pink stripe. The gradient red to green coloration of individual gene plots indicates values above or below the mean of the cluster, respectively. The cluster template profile is designated by a yellow line.

We used the Pavlidis Template Matching (PTM) algorithm [24], to divide the 4,151 TFR Unigenes into twenty gene groups or clusters. Specifically, twenty gene profiles of interest were selected [Additional File 4] to reflect major transcriptional patterns of development across E-L stages 31 to 38 (Figure 2). The PTM algorithm then classified the gene profiles into twenty groups via measurements of Pearson correlation: a correlation coefficient of greater than 0.75 was used to determine cluster membership. Six profiles showed a steady decline (profile groups 1 to 3) or increase (profile groups 9 to 11) in steady-state transcript abundance over time with distinctly different slopes. These six profile groups encompassed 63% of the Unigenes with a majority expressed in profiles 2 and 3 (31.9%) and profiles 9 and 11 (28%; Figure 2). Eight profiles had transient peak increases (profile groups 4 to 8) or decreases (profile groups 12 to 16) in transcript abundance at each of E-L stages 32 to 36. These transient profiles accounted for 22% of the Unigenes. A majority (68.2%) of these transiently expressed genes (profile groups 4 to 8 and 12 to 16) exhibited increased transcript abundance with the highest proportion within profile group 16 (E-L stage 36), followed closely by profile group 15 (E-L stage 35 around véraison), and profile group 12 (E-L stage 32) (Figure 2). Interestingly, genes with transient decreases early in berry development (profile groups 4 and 5) also exhibited large increases in transcript abundance during the later stages (E-L stages 36 to 38). The last four profiles (profile groups 17–20) were selected as having two peaks of expression between E-L stages 32 and 36 (Figure 2). Approximately 4.3% of transcripts had such "up and down" expression patterns (profile groups 17–20). Finally, Unigenes that did not match one of these profiles were grouped into a 21^st ^cluster (Figure 2), accounting for 11% of the total transcripts considered (profile group 21). Taken together, this analysis revealed that berry development is not only a progressive process, wherein the majority of genes exhibit a steady increase or decrease in expression across all stages of development (profile groups 1 to 3 and 9 to 11), but also a dynamic process, wherein a large number of genes exhibit large, transient changes in transcript abundance at specific times of development. Most notably, the last phase of berry development (Phase III, profile groups 14, 15 and 16) was the time when the largest number of genes (380 transcripts or 9.1%) exhibited transient increases in steady-state transcript abundance.

### Functional categorization of Unigenes across different stages of development

**Figure 3:**
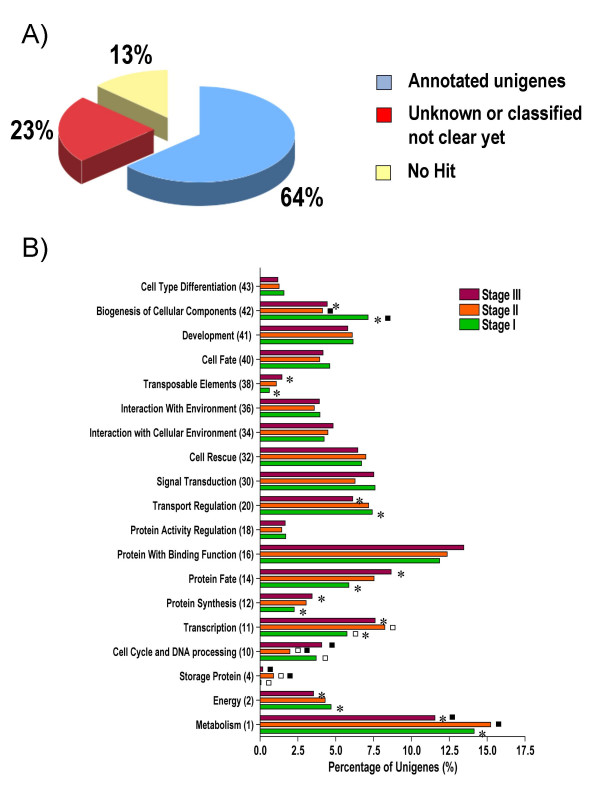
**Functional analyses of steady-state transcripts with a two-fold or greater change in abundance over the course of berry development**. A) Percentage of annotated unigenes with a two-fold or greater change in transcript abundance. B) Distribution of Unigenes according to their MIPS functional categories (MIPS 2.0) within the three main phases of berry development. Phase I (E-L stage 31), herbaceous phase; Phase II (E-L stages 32 to 34), lag phase; Phase III (E-L stages 35 to 38), ripening phase. Statistically significant differences between Phase I against II are indicated with white squares. Statistically significant differences between Phase II against III are indicated with black squares. Statistically significant differences between Phase I against III are indicated with asterisks. Percentages are based upon the number of Unigenes in each set. Numbers in parentheses following category names indicates the MIPS number for each category.

Functional categories were assigned to Unigenes with two-fold or greater changes in steady-state transcript abundance over the course of the seven developmental stages using the Munich Information Center for Protein Sequences (MIPS, ver. 2.0) catalog with annotations of the top Arabidopsis BLAST hits [25]. Because we detected some errors in the functional annotation for some Unigenes, functional categorization of each Unigene were verified manually and corrected if necessary. Corrections were only performed for the 4,151 Unigenes that displayed a two-fold or greater change in expression [See Additional File 2: Table 4]. Functional annotations could be assigned to approximately 64% of transcripts (Figure 3A). An additional 23% of Unigenes had matches to genes with unknown functions or unclear classifications (unclassified), and 13% did not have a BLAST hit (no hit) in public, non-redundant (NR) databases. The relative distribution of Unigenes within each of nineteen functional categories was determined (Figure 3B). To facilitate a functional comparison of the three major stages of berry development, Unigenes from each of the profile groups were regrouped into the three major developmental phases to reflect the greatest degree of transcript abundance changes at each phase: Phase I (profiles 1, 2, and 3), Phase II (profiles 4, 5, 6, 12, 13, and 14), and Phase III (profiles 7, 8, 9, 10, 11, 15, and 16). Statistically significant differences in the distribution of genes within functional categories amongst these developmental stages were observed (Figure 3B; see Additional File 5: Tables 1 and 2). Functional categories that had a large number of transcripts in Phase I followed by a decrease in Phase III included biogenesis of cellular component (42), transport regulation (20), energy (2), and metabolism (1). This is consistent with the developmental aspects of this phase, which are characterized by cell division and expansion, which require a high level of metabolic activity. The process of cell division requires large quantities of structural materials and consumes energy, while cell expansion requires large quantities of solutes and water.

The opposite trend of increasing transcript abundance from Phase I to Phase III was observed for functional groups that included transcription (11), protein synthesis (12), protein fate (14), protein with binding function (16), and to a lesser extent with interaction with cellular environment (34). These trends served to further indicate the complexity of the transcriptional, translational, and interaction-based regulatory processes necessary for berry development.

### Exemplar Unigenes associated with important molecular events of berry development

**Figure 4:**
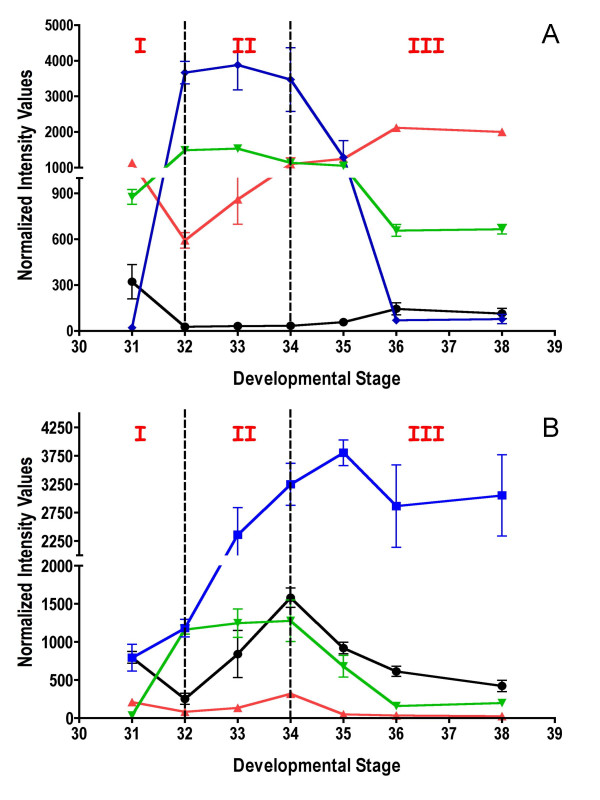
**Transcripts displaying transient expression patterns**. Each value plotted is the mean normalized intensity values obtained for the three biological replicates. The three key phases of the berry development (I, II, III) were applied as reference. **A) **Black solid round (1618814_at, NP864096)-ornithine decarboxylase, red solid triangle (1616399_s_at, CB005833)-arginine decarboxylase, green solid triangle (1611257_a_at, TC51832)-L-asparaginase, blue solid diamond (1618848_at, TC52577)-xyloglucan endotransglycosylase transferase. **B) ** Black solid round (1608074_s_at, TC62965)-α-expansin, red solid triangle (1608191_at, TC64448) α-expansin, green solid triangle (1613161_at, TC69794)-limonene cyclase, blue solid diamond (1618595_at, TC53841)-(-)-isopiperitenol dehydrogenase.

In order to identify genes with potentially important roles in specific stages of berry development, transcripts with a dynamic pattern were identified from within the first 20 PTM algorithm-defined profile groups. The transcript profiles were examined in further detail (Figure 4).

Polyamines (PAs) are a class of compounds that have plant growth regulator activity. Their roles in cell division [26] and fruit set [27] have been widely investigated. Free, conjugated and wall-bound forms of polyamines accumulate mostly at anthesis before decreasing at fruit set in grapes [28]. Two transcripts were detected that belong to profile 4, which are strongly down-regulated at E-L stage 32 (1618814_at, 1616399_s_at; AY174164, TC68466). Both are related to ornithine decarboxylase and arginine decarboxylase, which are involved in polyamine metabolism [29]. These two genes located at the start of the PA pathway might play a role in providing precursors that would be used during Phase I of berry development.

In higher plants, the catabolism of asparagine (Asn) occurs by two routes. The first pathway involves the hydrolysis of Asn, releasing ammonia and aspartate by asparaginase activity. L-asparaginase is one of the enzymes for Asn utilization by plants that plays an important role in the nitrogen metabolism of developing plant tissues [30]. One Unigene encoding L-asparaginase (1611257_a_at; TC51832) displayed a specific peak during E-L stages 32 (Figure 4A). This last result indicates that this enzyme could play a role during the first phase of berry development as a provider of ammonia for *de novo *protein synthesis in grape. This result is also supported by the significant transcript abundance of Unigenes encoding glutamate dehydrogenase or glutamine synthetase (data not shown, see Additional File 2: Table 4, 1607579_at, 1613697_at, 1609819_s_at) during the first phase of berry development. These enzymes participate in nitrogen assimilation in plants [31].

In grape berries, fruit softening occurs during Phase III and is largely affected by cell-wall loosening [32] and turgor [33]. Xyloglucans account for about 10% of the cell wall composition in berries [32]. In fruit, xyloglucan depolymerization is associated with fruit softening [34]. Xyloglucan endotransglycosylases, which hydrolyze and transglycosylate xyloglucans, were encoded by multiple isogenes, the majority of which were expressed highly during Phase I in berry development (E-L stage 31), but then declined (data not shown; see Table 1). One Unigene (1618848_at; TC52577), however, which is a xyloglucan endotransglucosylase/hydrolase, displayed a 185-fold increase in expression during Phase II, peaking at E-L stage 33 (Figure 4A). This xyloglucan endotransglucosylase Unigene is closely related to a xyloglucan endotransglucosylase/hydrolase (SIXTH5) that can act reversibly. It has been characterized recently as a tomato xyloglucan depolymerase *in vitro *in the presence of xyloglucan oligosaccharides (XGOs) [35].

**Table 1:** Transcripts (TFR pool) related cell wall metabolism categorized by the first hit in the MIPS2 catalog

**Probeset ID**	**GenBank Annotation**	**VvGI5**	**UniProt ID**	**Gene Name Description**	**Function**	**Profile**	**Fold Change**
1622791_at	CB973455	TC56114	Q6J8X2	Cellulose synthase	Cell Wall Biosynthesis	2	112.68
1619280_at	CF211860	TC59569	Q6J8W9	Cellulose synthase	Cell Wall Biosynthesis	2	87.25
1613018_at	CB971117	TC61561	Q851L8	Cellulose synthase	Cell Wall Biosynthesis	2	6.92
1606646_at	CA812296	TC56773	Q6XZC2	Cellulose synthase	Cell Wall Biosynthesis	2	4.34
1615577_at	CB340193	TC52068	Q6XP46	Cellulose synthase	Cell Wall Biosynthesis	3	3.5
1607069_at	CB982496	TC53451	Q45KQ0	Cellulose synthase	Cell Wall Biosynthesis	10	3.18
1611149_at	BM437543	TC56091	Q3Y6V1	Cellulose synthase	Cell Wall Biosynthesis	21	2.85
1612999_at	CF513786	-	O80890	Cellulose synthase	Cell Wall Biosynthesis	7	2.76
1620206_at	CF515519	TC66132	Q6FD0	β 1,4-Mannan synthase	Cell Wall Biosynthesis	15	2.69
1616808_at	CF207742	TC57597	Q45KQ0	Cellulose synthase	Cell Wall Biosynthesis	21	2.21
1619938_at	CF514664	TC63356	Q6YBV2	Cellulose synthase	Cell Wall Biosynthesis	3	2.19
1620840_at	CB968965	TC53122	Q4F8K2	α-expansin	Cell Wall Expansion	2	20.26
1619010_s_at	BQ794765	TC54832	Q84US9	Expansin	Cell Wall Expansion	10	18.54
1608191_at	CD798831	TC64448	Q49QW6	Expansin	Cell Wall Expansion	21	13.18
1612253_at	CB970527	TC62108	Q6RX68	Expansin	Cell Wall Expansion	3	9.23
1608074_s_at	CF215793	TC62965	Q84UT0	Expansin	Cell Wall Expansion	21	6.28
1610418_at	BQ798078	TC67284	Q8GZD3	Expansin	Cell Wall Expansion	10	4.9
1613527_at	CB978490	TC53065	Q6T5H5	Expansin	Cell Wall Expansion	15	4.6
1618121_at	CF213691	-	Q9LUI1	Extensin	Cell Wall Expansion	2	4.1
1608504_at	BQ797231	TC52168	Q6K4C6	Expansin	Cell Wall Expansion	4	2.56
1612154_at	CB970048	TC61667	O50044	KDO 8-P synthase	Cell Wall Expansion	3	2.31
1609651_at	CF404678	TC55463	Q9LJX2	Pectinesterase inhibitor	Cell Wall Modification	2	690.87
1618848_at	CB977336	TC52577	Q9ZRV1	Xyloglucan endotransglycosylase 1	Cell Wall Modification	13	184.42
1622288_at	CB974798	TC59058	Q9M660	Cell-Wall P4	Cell Wall Modification	2	124.19
1617556_s_at	BQ797260	TC67257	Q9M4I1	Proline-rich cell wall protein	Cell Wall Modification	10	105.38
1619762_at	CF214586	TC67718	Q7Y250	Arabinogalactan protein	Cell Wall Modification	2	61.79
1620201_at	CB972625	TC70982	Q53WM8	Pectinesterase	Cell Wall Modification	2	42.57
1619519_at	CB971445	TC65487	Q7Y250	Arabinogalactan protein	Cell Wall Modification	2	39.53
1616045_a_at	AJ237983	-	Q9M4I0	Proline-rich cell wall protein	Cell Wall Modification	11	38.03
1617023_at	CF210510	TC53552	FLA1	Arabinogalactan protein	Cell Wall Modification	3	37.53
1611601_at	CB977009	TC57247	Q6ZDX2	Pectinesterase	Cell Wall Modification	2	34.83
1619613_at	CD801720	TC68597	Q9SAP3	Proline-rich protein	Cell Wall Modification	2	34.56
1616528_s_at	CD801342	TC55188	Q1SAY6	Proline-rich protein	Cell Wall Modification	2	33.73
1621880_s_at	CK138206	TC66098	Q8VZG5	β-xylosidase	Cell Wall Modification	3	31.38
1608727_at	CB973483	TC56396	Q9LZX4	Fasciclin arabinogalactan protein 10	Cell Wall Modification	3	30.04
1615533_s_at	CF415374	TC51824	Q7Y252	Endo-xyloglucan transferase	Cell Wall Modification	3	27.95
1622481_x_at	CF568921	TC67150	Q39763	Proline-rich protein	Cell Wall Modification	1	27.63
1614426_at	CD801116	TC64184	Q4F8J3	Xyloglucan endotransglycosylase	Cell Wall Modification	3	25.94
1619522_at	AY043231	TC56838	Q94B17	β-galactosidase	Cell Wall Modification	3	24.53
1622292_at	CF403386	TC69174	Q949Z1	polygalacturonase	Cell Wall Modification	2	24.24
1622295_at	CF215662	TC68541	Q5CCP8	β-galactosidase	Cell Wall Modification	3	24.1
1621477_s_at	CF215974	TC67884	Q9LYT5	Pectinesterase	Cell Wall Modification	1	23.62
1622121_at	BQ799039	TC58094	Q4F8J0	Cellulase	Cell Wall Modification	3	22.85
1615201_at	CF512517	TC63907	Q96232	Proline-rich-like protein	Cell Wall Modification	3	22.42
1618657_at	CF211626	TC56055	Q84LI7	Exopolygalacturonase	Cell Wall Modification	2	20.99
1616158_at	CD801717	TC53176	Q4JLV6	Pectate lyase	Cell Wall Modification	21	20.15
1612239_at	CF610039	TC55421	Q8VZG5	β-xylosidase	Cell Wall Modification	2	19.96
1620140_at	CF208989	TC53499	Q40161	Polygalacturonase	Cell Wall Modification	2	19.6
1611747_at	CF608890	TC65113	Q7XAS3	β-D-glucosidase	Cell Wall Modification	3	17.94
1609909_s_at	CF206328	TC64184	Q4F8J3	Xyloglucan Endotransglycosylase	Cell Wall Modification	3	15.14
1608313_at	CF209144	TC52275	Q76MS4	β-xylosidase	Cell Wall Modification	2	14.41
1615574_at	CB977067	TC56317	Q9M5J0	Pectinesterase	Cell Wall Modification	1	14.03
1619612_at	CF211611	TC67414	Q94KD8	β-xylosidase	Cell Wall Modification	2	13.83
1610073_at	CF206157	TC51796	Q8S902	Xyloglucan Endotransglycosylase	Cell Wall Modification	3	13.77
1621251_s_at	BQ795002	TC69305	Q8W3L8	Xyloglucan Endotransglycosylase 2	Cell Wall Modification	10	13.64
1622735_s_at	CB340122	TC51796	Q84JX3	Xyloglucan Endotransglycosylase	Cell Wall Modification	3	13.47
1613844_at	CF404099	TC54968	Q9LUG8	Endo-1,3-1,4-β-D-glucanase	Cell Wall Modification	3	13.27
1617755_at	CF213513	TC52924	Q8GSQ4	Pectin-glucuronyltransferase	Cell Wall Modification	2	12.86
1615746_at	CB970034	TC53433	Q9FXI9	Endo-1,4-β-glucanase	Cell Wall Modification	3	11.99
1617785_at	CD800122	TC54681	Q9LW90	Pectinesterase	Cell Wall Modification	3	11.97
1607374_at	CF404162	TC69448	Q7XAS3	β-D-glucosidase	Cell Wall Modification	3	11.77
1610311_at	CF373485	TC52429	Q41725	Arabinogalactan protein	Cell Wall Modification	3	10.99
1620096_at	CF372841	TC57673	Q4F986	Xyloglucan endotransglycosylase	Cell Wall Modification	2	10.81
1616093_at	CF404665	TC69415	Q7XA92	Pectinesterase	Cell Wall Modification	3	10.36
1613467_at	CF212805	TC54247	Q9FSW6	Arabinogalactan protein	Cell Wall Modification	15	10.08
1617875_at	CB971740	TC61493	O04477	β-N-acetylhexosaminidase	Cell Wall Modification	3	9.73
1614803_at	AY046416	TC70108	Q8LGR6	Proline-rich protein	Cell Wall Modification	3	9.22
1616822_at	AF220196	TC70108	Q8LGR6	Proline rich protein	Cell Wall Modification	3	9.04
1610756_at	CF604824	TC55088	Q9LT39	Polygalacturonase inhibitor	Cell Wall Modification	1	8.2
1622591_at	CB981129	TC70200	Q9FHN6	Pectinesterase	Cell Wall Modification	2	8.06
1612672_at	CF215975	TC62593	Q9SEE7	Pectinesterase	Cell Wall Modification	2	7.61
1616522_at	CF403905	TC55346	Q9LEB0	Pectinesterase	Cell Wall Modification	2	7.51
1615198_at	CF209943	TC65883	Q9LEC9	β-xylosidase	Cell Wall Modification	3	7.48
1608756_at	BQ798436	TC59719	Q84LI7	Polygalacturonase	Cell Wall Modification	2	7.15
1609790_at	CF207994	TC55069	Q4F8J3	Xyloglucan endotransglycosylase	Cell Wall Modification	2	6.87
1614877_at	CB002982	TC66230	Q9C8T5	Proline-rich protein	Cell Wall Modification	2	6.78
1613330_at	CF404655	-	Q93Z77	Pectate lyase	Cell Wall Modification	3	6.64
1608120_at	CF603941	TC70545	Q6U6I9	Pectate lyase	Cell Wall Modification	2	6.6
1613677_at	CB969707	TC51953	Q6J192	Arabinogalactan protein	Cell Wall Modification	2	6.16
1619383_s_at	BQ794831	TC66587	Q5CCQ0	β-galactosidase	Cell Wall Modification	3	6.14
1615603_at	CB346190	TC64570	Q8VY93	Proline-rich protein	Cell Wall Modification	3	5.75
1608180_at	CF201469	TC68224	O23950	Endo-xyloglucan transferase	Cell Wall Modification	2	5.51
1609593_at	CB981468	TC68226	Q9LZV3	(1-4)-β-mannan endohydrolase	Cell Wall Modification	15	5.49
1621225_at	CB974537	TC52140	Q9SUP5	Polygalacturonase	Cell Wall Modification	21	5.12
1613415_at	AB074999	TC45132	Q8W3L8	Xyloglucan endotransglycosylase 1	Cell Wall Modification	10	5.1
1615995_at	CF212592	-	P24806	Xyloglucan Endotransglucosylase 24	Cell Wall Modification	21	5.02
1615809_at	CB980277	TC69342	Q38908	Xyloglucan endotransglucosylase 30	Cell Wall Modification	11	4.87
1613719_at	CF214562	TC69710	Q7Y250	Arabinogalactan protein	Cell Wall Modification	2	4.8
1613528_at	CF513262	TC66769	Q8LPS9	Pectinesterase	Cell Wall Modification	2	4.56
1612668_at	CF519076	TC61610	Q5CHL3	Hydroxyproline-rich glycoprotein	Cell Wall Modification	21	4.32
1620063_at	CB921343	TC61082	Q9M3U4	β-1-3 glucanase	Cell Wall Modification	11	4.3
1611233_at	CF605724	TC66632	Q4W7I3	β-xylosidase	Cell Wall Modification	3	4.18
1622770_at	CF209970	TC66250	O65186	Cellulase	Cell Wall Modification	13	4.15
1609653_at	BQ797078	TC70494	Q9SBM1	Hydroxyproline-rich glycoprotein	Cell Wall Modification	10	4.15
1620618_at	BQ794587	TC55377	Q8LAB2	Proline-rich protein	Cell Wall Modification	2	3.59
1608799_at	BQ800204	TC58800	Q4ABV3	Pectinesterase	Cell Wall Modification	3	3.55
1616523_s_at	CF512513	TC63963	Q8L9T8	Arabinogalactan protein	Cell Wall Modification	1	3.53
1606652_at	CB969544	TC52628	Q8H1N7	Polygalacturonase	Cell Wall Modification	2	3.52
1622353_at	BQ800489	TC51768	Q5TIN5	β-6-xylosyltransferase	Cell Wall Modification	3	3.41
1619659_s_at	CF405842	TC68391	A1IIA8	Pectate lyase	Cell Wall Modification	14	3.37
1617920_at	CF609275	TC52380	Q6QLN2	Endo-1,4-β-glucanase	Cell Wall Modification	2	3.31
1608896_at	BQ796455	TC59657	Q5BM98	Secondary cell wall-related glycosyltransferase	Cell Wall Modification	4	3.24
1618849_at	BQ799201	TC63732	Q9SUP5	Polygalacturonase	Cell Wall Modification	21	3.16
1610996_at	BQ794786	TC63941	Q43111	Pectinesterase 3	Cell Wall Modification	14	3.15
1615125_at	CF372050	TC67073	Q5BM97	Secondary cell wall-related glycosyltransferase family 14	Cell Wall Modification	2	3.09
1608945_at	BQ793580	TC54729	P35694	Xyloglucan endotransglycosylase	Cell Wall Modification	15	3.09
1607567_at	BQ795116	TC54314	Q564G6	Galactomannan galactosyltransferase	Cell Wall Modification	11	3.06
1619068_at	CF215954	TC60314	Q94B11	Xyloglucan endotransglycosylase	Cell Wall Modification	3	2.78
1612425_at	CF371700	TC56348	Q6EP64	Hydroxyproline-rich glycoprotein	Cell Wall Modification	11	2.77
1616826_at	CB976610	TC54888	Q599J2	β-1,2 Xylosyltransferase	Cell Wall Modification	11	2.76
1609138_at	CF519079	TC66620	Q16861	Super cysteine rich protein	Cell Wall Modification	11	2.74
1617487_at	CD720403	TC54500	Q9SFF6	Pectinacetylesterase	Cell Wall Modification	2	2.69
1617687_at	CB981123	TC57577	Q494P2	Xyloglucan endotransglycosylase 2	Cell Wall Modification	21	2.67
1606832_at	CF214798	TC51861	Q7Y223	(1-4)-β-mannan endohydrolase	Cell Wall Modification	2	2.58
1617712_at	CF607664	TC67150	Q39789	Proline-rich cell wall protein	Cell Wall Modification	2	2.52
1617919_at	CF605842	TC55276	Q9SHZ2	β-1,3-glucanase	Cell Wall Modification	18	2.4
1617015_at	CF209172	TC54616	Q7XRM8	Pectate lyase	Cell Wall Modification	2	2.34
1618863_at	CF208339	TC52953	Q93Y12	α-glucosidase	Cell Wall Modification	3	2.28
1617939_s_at	CB910883	TC52435	Q41695	Pectinacetylesterase	Cell Wall Modification	1	2.28
1616734_at	CF405846	TC52115	Q6ZIF8	Pectin-glucuronyltransferase	Cell Wall Modification	3	2.28
1607945_at	AF243475	-	Q9M505	Pectate lyase	Cell Wall Modification	2	2.27
1612551_at	CF605967	TC63126	Q9M3C5	β-N-acetylhexosaminidase	Cell Wall Modification	21	2.26
1622843_s_at	CF212102	TC65557	Q9LVC0	Arabinogalactan protein	Cell Wall Modification	4	2.25
1611230_at	AF159124	-	Q9XGT3	β-galactosidase	Cell Wall Modification	2	2.25
1619468_at	AY043232	TC38735	Q94B16	Pectin methylesterase PME1	Cell Wall Modification	12	2.24
1610118_at	CB974025	TC60557	O23562	Glucanase	Cell Wall Modification	18	2.21
1614868_at	CB920940	TC64720	Q9M0S4	Arabinogalactan protein	Cell Wall Modification	5	2.17
1607528_at	AY043236	TC61627	Q94B12	Cellulase CEL1	Cell Wall Modification	21	2.11
1614814_s_at	CB345895	TC57381	O24136	CP12 precursor	Cell Wall Modification	13	2.07

Expansins play important roles in cell wall loosening via non-enzymatic mechanisms and are involved in cell expansion [36]. Most expansin genes displayed steadily increasing or decreasing patterns during berry development (see Table 1). Others showed peak expression around E-L stage 34 (α-expansin, 1608074_at, TC62965; α-expansin, 1608191_at, TC64448; Figure 4B). An expansin gene from strawberry, *FaExp4*, displays exactly the same peak transient expression pattern as these latter two genes at a comparable ripening stage as grape berries, called the White stage in strawberry fruits, just before red fruit color development [37]. Thus, these expansins in grape berry may be required during the Phase III of grape berry development, when the second phase of cell expansion occurs.

Terpenes, which are precursors for important aroma compounds [38], accumulate at véraison [39,40]. One Unigene encoding a limonene cyclase (1613161_at; TC69794; Figure 4B), which is in the monoterpene pathway, is involved in the conversion of geranyl diphosphate into limonene [41]. Limonene and some of its derived compounds such as menthol or 1,8 cineol are intimately associated with the "eucalyptus fragrance" of red wine [42]. Accumulation of 1,8-cineole in wines is derived from precursors in grape, like limonene. The strong induction of our Unigene related to limonene cyclase (~40 fold from E-L stages 32 to 34) correlates well with the beginning of accumulation of 1,8-cineole in red grape samples [43]. One Unigene (1618595_at, TC53841; Figure 4B) belonging to profile 15 and encoding alcohol dehydrogenase exhibited strong homology with an (-) isopiperitenol dehydrogenase, which is involved in the same monoterpene pathway [44]. This transcript abundance of this Unigene is correlated to the expression of the limonene cyclase previously discussed above indicating a possible activation of these enzymes in the same metabolic pathway [44].

### Phytohormone biosynthesis and responses

**Figure 5:**
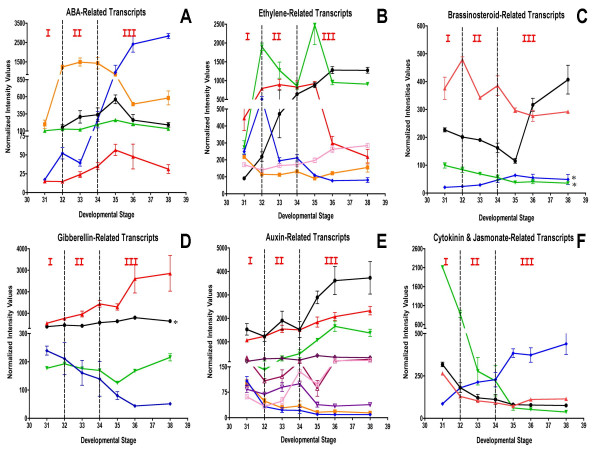
**Expression of phytohormone transcripts**. **A) **Black solid round (1608022_at, TC57089)-NCED isoform 1, red solid triangle (1607029_at, TC55541)-NCED isoform 4, green solid triangle (1614892_at, TC54474)-ABI1 protein phosphatase type 2C, blue solid diamond (1619802_at, TC67323)-RD22, orange solid square (1621346_at, TC65114)-ABI3 transcription factor. **B) **Black solid round (1617012_at, TC68057)-ethylene responsive factor 1, red solid triangle (1619585_at, TC62897)-ethylene induced transcription factor, green solid triangle (1621552_at, TC66829)-ethylene co-activator, blue solid diamond (1615952_s_at, TC56709)-aminocyclopropane carboxylic acid synthase, orange solid square (1622402_at, TC62349)-ERS1 ethylene receptor, lavender open square (1618518_at, TC55908)-EIN4/ETR5 ethylene receptor. *: transcript that does not pass the two-fold ratio. **C) **Black solid round (1617572_at, TC66046)-BRH1 brassinosteroid-responsive protein, red solid triangle (1612516_at, TC56501)-BRI1 brassinosteroid-responsive protein, green solid triangle (1619068_at, TC60314)-brassinosteroid-responsive protein, blue solid diamond (1608945_at, TC54729)-BRU1 brassinosteroid-responsive protein. *: transcript that does not pass the two-fold ratio. Black solid round (1618181_at, TC67464)-GIDL1 receptor, red solid triangle (1620071_at, TC56624)-GIDL2 Receptor, green solid triangle (1606777_s_at, TC56894)-GA1a gibberellin oxidase, blue solid diamond (1610610_at, TC66284)-gibberellic acid β hydroxylase. *: transcript that does not pass the two-fold ratio. **E) **Black solid round (1614660_at, TC53887)-auxin responsive protein (Aux22), red solid triangle (1613813_a_at, TC65541)-auxin responsive factor 2, green solid triangle (1609591_at, TC63193)-small auxin up RNA protein, blue solid diamond (1606566_at, TC62299)-SAUR protein, orange solid square (1616225_at, TC52772)-auxin responsive factor 18, lavender open square (1619610_at, TC56575)-IAA-amino acid hydrolase, brown open triangle (1611479_at, CD799903)-auxin transporter, pink open triangle (1617179_at, CF414958)-auxin efflux carrier, purple open diamond (1610034_at, TC59892)-auxin binding protein. **F) **Black solid round (1607601_at, TC61395)-12-oxophytodienoate reductase, red solid triangle (1614324_at, CF213899)-constitutive pathogen response 5 (CPR5), green solid triangle (1620306_at, TC69712)-cytokinin oxidase, blue solid diamond (1612955_at, TC52530)-Type-A response regulator.

A number of plant growth regulators including abscisic acid (ABA), auxin (indole-3-acetic acid [IAA], brassinosteroids (BR), ethylene, and gibberellic acid (GA) have been implicated in the control of berry development and ripening. Therefore, steady-state transcript accumulation patterns of Unigenes with functions related to hormone biosynthesis and response were tracked over the course of berry development (Figure 5, Table 2).

**Table 2:** Transcripts (TFR pool) related to phytohormone biosynthesis and response categorized by the first hit in the MIPS2 catalog

**Probeset ID**	**GenBank Annotation**	**VvGI5**	**UniProt ID**	**Gene Name Description**	**Function**	**Profile**	**Fold Change**
1608022_at	BQ798105	TC57089	Q5SGD1	9-cis-epoxycarotenoid dioxygenase 1	ABA biosynthesis	15	6.46
1607029_at	CD716868	TC55541	Q8LP14	9-cis-epoxycarotenoid dioxygenase 4	ABA biosynthesis	15	3.85
1617541_s_at	CB342503	TC54423	O49814	β-carotene hydroxylase 2	ABA biosynthesis	3	3.25
1618171_s_at	BQ792407	TC55939	Q5SGC9	Zeaxanthin epoxidase	ABA metabolism	3	2.36
1614788_at	BQ792954	TC54112	Q3ZNL4	Dehydrin 1a	ABA response	11	26.63
1609063_at	BQ799245	TC63341	Q4VT47	RD-22 (ABA regulated)	ABA response	3	11.28
1621346_at	CB978597	TC65114	O48620	ABI3 (ABA regulated)	ABA response	14	7.57
1614892_at	CF511230	TC54474	O82468	Phosphatase 2C (ABA regulated)	ABA response	11	5.87
1615970_at	CF405892	TC65344	Q7XAV5	Dehydration responsive element binding protein	ABA response	15	4.53
1616735_at	CF604749	TC51916	O82176	Phosphate-induced protein (ABA regulated)	ABA response	16	3.44
1607955_at	CB978189	TC63879	Q9ZST5	PII protein (ABA regulated)	ABA response	12	3.07
1621396_at	CF514715	TC51970	Q94IB2	Phi-2 (ABA regulated)	ABA response	16	2.9
1617417_s_at	CD798528	TC61938	Q9M3V0	Phosphatase 2C (ABA regulated)	ABA response	5	2.77
1617791_s_at	CB004910	TC70554	Q45W74	Dehydration-induced protein (ABA regulated)	ABA response	4	2.55
1609665_a_at	CB005515	TC58443	Q9M9W9	Phosphatase-2C (ABA regulated)	ABA response	20	2.25
1609419_at	CB982969	CB982969	Q9S7V4	Abscisic acid-induced protein	ABA response	15	2.23
1610937_at	BQ792881	TC65459	Q67WL5	Abscisic acid-induced protein	ABA response	18	2.2
1611714_at	BQ794807	TC53528	Q06009	Phosphatase 2A (ABA regulated)	ABA response	3	2.12
1616882_at	CD799018	TC53254	Q7Y0S8	Phi-1 (ABA regulated)	ABA response	3	2.1
1621041_at	BQ794656	TC56829	Q9FIE3	Vernalization-insensitive protein 3 (ABA regulated)	ABA response	21	2.08
1619261_s_at	CB982969	TC68788	Q5XWP1	Abscisic acid-induced protein	ABA response	21	2.05
1619272_at	CF373376	TC51939	Q94AL8	Cold acclimation protein (ABA regulated)	ABA response	3	2.02
1619610_at	CB008850	TC56575	Q84XG9	IAA-amino acid hydrolase	Auxin metabolism	11	5.86
1615645_at	CB969433	TC62316	Q8LCI6	IAA-amino acid hydrolase	Auxin metabolism	3	3.19
1606566_at	CF211641	TC62299	Q681Q1	Auxin-induced protein	Auxin response	2	12.05
1609591_at	CD799271	TC63193	O23089	Auxin-regulated protein	Auxin response	11	11.74
1614851_s_at	CB973279	TC62879	Q1RY17	Auxin responsive Factor	Auxin response	3	11
1620662_at	CB981820	TC59676	Q6QUQ3	Auxin and ethylene responsive GH3	Auxin response	11	10.17
1614098_at	CF608417	TC57853	Q9FEL8	AUX1 like protein (influx carrier)	Auxin response	2	8.8
1606509_at	CB971327	TC52521	Q7XEJ9	Auxin induced protein	Auxin response	3	8.29
1616225_at	CB972698	TC52772	Q9C5W9	Auxin response factor 2	Auxin response	1	7.3
1612060_at	CB346335	TC53973	Q76DT1	AUX1 like protein (influx carrier)	Auxin response	7	6.96
1616104_at	CB004955	TC55019	O65695	Auxin-regulated protein	Auxin response	15	6.11
1612001_s_at	CF604676	TC69850	Q9XEY0	Nt-gh3 (auxin and ethylene)	Auxin response	2	4.69
1617163_at	BQ800616	TC56821	Q9SHL8	Auxin efflux carrier	Auxin response	3	4.23
1617513_at	CF203551	TC52262	Q8LAL2	Auxin-responsive protein IAA26	Auxin response	2	3.94
1613054_at	BQ794856	TC53877	O65695	Auxin regulated factor	Auxin response	3	3.79
1621946_at	CB975415	TC70724	Q8H0E0	PIN1 like auxin transport	Auxin response	7	3.7
1616015_at	CF607669	TC67186	Q2LAJ4	Auxin response factor	Auxin response	7	3.69
1611491_at	CB900901	TC57901	Q769J4	AtPIN3 (auxin efflux carrier)	Auxin response	20	3.5
1612180_at	CF608682	TC66988	Q6L8T9	Auxin responsive factor 5	Auxin response	7	3.49
1614660_at	CF207466	TC53887	P13088	Auxin-induced protein AUX22	Auxin response	11	3.04
1617179_at	CF414958	-	Q6YZX7	Auxin efflux carrier	Auxin response	21	2.91
1615728_at	AY082522	TC60981	Q84V38	CsIAA3 (Auxin regulated)	Auxin response	7	2.66
1610034_at	CB97302	TC59892	Q49RB8	Auxin receptor	Auxin response	10	2.45
1617097_at	BQ797969	TC67796	Q8LER0	Auxin efflux carrier	Auxin response	3	2.41
1613857_at	CD715051	TC55162	O24408	Auxin responsive factor	Auxin response	2	2.32
1607503_s_at	CF515267	TC53837	Q52QX4	Auxin-repressed protein	Auxin response	12	2.3
1619658_at	CF371851	TC66647	Q6QUQ3	Auxin and ethylene responsive GH3	Auxin response	15	2.25
1610591_at	CB923320	TC57860	Q3LFT5	Auxin regulated protein	Auxin response	10	2.21
1620726_at	CB339504	TC56077	Q6YZJ0	Auxin-regulated protein	Auxin response	3	2.19
1617694_at	CB972462	TC62076	Q93XP5	Auxin responsive factor	Auxin response	2	2.18
1618394_at	CF371644	CF371644	Q949J8	Auxin growth promoter protein	Auxin response	2	2.1
1617572_at	CB918599	TC66046	Q9XF92	BRH1 RING finger protein (Brassinosteroid regulated)	Brassinosteroid response	21	3.53
1620306_at	CF404552	TC69712	Q5ZAY9	Cytokinin oxidase	Cytokinin response	3	57.36
1610071_at	BQ797708	TC58750	Q39802	Cytokinin induced message	Cytokinin response	11	13.16
1619945_at	CB345883	TC61250	Q84N27	Cytokinin repressed protein	Cytokinin response	19	3.05
1622308_at	CF210289	TC63310	Q8S933	1-aminocyclopropane-1-carboxylate synthase	Ethylene biosynthesis	2	11.84
1615952_s_at	CF215641	TC56709	Q84X67	1-aminocyclopropane-1-carboxylic acid oxidase	Ethylene biosynthesis	3	6.56
1609683_at	CF604955	TC65735	Q5U8L6	Ethylene responsive factor 2	Ethylene response	12	22.64
1617012_at	CD802399	TC68057	P16146	Ethylene-responsive element	Ethylene response	11	14.05
1621552_at	BM437510	TC66829	Q9LV58	Ethylene-responsive transcriptional co activator	Ethylene response	21	9.08
1619585_at	CD800299	TC62897	Q75UJ4	Ethylene responsive factor	Ethylene response	13	4.21
1611910_s_at	AY395745	TC63214	Q6TKQ3	Ethylene responsive factor 4	Ethylene response	3	4.13
1609990_at	CB009298	TC63214	Q6TKQ3	Ethylene responsive factor 2	Ethylene response	3	4.12
1608511_at	CB342877	TC62587	Q6RZW7	Ethylene responsive factor 5	Ethylene response	15	3.72
1609780_at	CA810742	TC55438	Q94E74	Ethylene responsive factor 6	Ethylene response	3	3.22
1612699_at	BQ798614	BQ798614	Q9XIA5	Ethylene-forming-enzyme-like dioxygenase	Ethylene response	12	2.75
1619178_at	CB349106	TC54200	Q94E74	Ethylene responsive 6	Ethylene response	19	2.62
1613799_at	CF517211	TC55673	Q6RZW8	Ethylene responsive factor 4	Ethylene response	21	2.5
1622402_at	CD799344	TC62349	Q84PH6	Ethylene receptor (EIN4)	Ethylene response	20	2.4
1609559_at	CF215263	TC58568	Q94AW5	Ethylene-responsive element	Ethylene response	21	2.34
1606623_at	BQ797592	TC70037	Q9LVS8	EREBP-4	Ethylene response	3	2.28
1612921_at	CF514773	TC57403	Q9LVS8	EREBP-4	Ethylene response	3	2.26
1618213_at	CF203873	CF203873	Q9SWV2	ER6 (Ethylene regulated)	Ethylene response	3	2.16
1611657_at	CF208861	TC67832	O64588	GH3 Root formation (gibberellin regulated)	GA response	3	8.07
1610607_at	CF371650	TC66111	Q49RB3	GASA	GA response	3	6.98
1621228_at	BQ798029	TC52322	Q6S5L6	GAI protein (Gibberellin regulated)	GA response	7	2.52
1610610_at	CA810332	TC66284	Q9ZQA5	Putative gibberellin β-hydroxylase	Gibberellin metabolism	10	3.52
1620071_at	BQ800214	TC56624	Q9LYC1	Gibberellin receptor	Gibberellin response	11	5.3
1618181_at	CF512673	TC67464	Q9MAA7	Gibberellin receptor 2	Gibberellin response	16	2.1
1622456_at	CF609276	TC66424	Q7PCB5	Phytosulfokine	Growth factor	3	9.3
1616312_at	CD720049	TC61028	Q7PCA0	Phytosulfokine peptide precursor	Growth factor	12	3.69
1607170_s_at	CB917184	TC66717	Q7PCA1	Phytosulfokine	Growth factor	21	2.07
1612021_at	CF213898	CF213898	Q7EYF8	Phytosulfokine receptor	Growth factor response	3	4.06
1607601_at	CF209956	TC61395	Q76DL0	12-oxophytodienoate reductase	JA metabolism	2	4.2
1619407_s_at	CA809049	TC67104	Q76DL0	12-oxophytodienoate reductase	JA metabolism	21	2.92
1620308_at	CF208037	TC57918	Q38944	Steroid 5-alpha-reductase	Lipid, fatty acid and isoprenoid metabolism	3	3.5
1613941_at	CA818531	TC61611	Q7X9G5	Lipoxygenase	Lipid, fatty acid and isoprenoid metabolism	1	2.99
1618940_at	CF212858	TC64939	Q8W250	1-deoxy-D-xylulose 5-phosphate reductoisomerase	Lipid, fatty acid and isoprenoid metabolism	3	2.03
1613678_at	CB971023	TC54495	Q9M2G7	Phosphatase	Phosphate metabolism	3	2.07
1612552_at	CA818350	CA818350	Q9C9W8	S-adenosyl-L-methionine:salicylic acid carboxyl methyltransferase	SA response	11	6.99
1618457_at	CF205125	CF205125	Q9M6E7	UDP-glucose:salicylic acid glucosyltransferase	SA response	12	2.19
1619377_at	CF372632	TC68498	Q5Z825	avrRpt2-induced AIG2 protein	SA response	12	2.06

### Abscisic acid

ABA amounts in berries decrease after anthesis, but then increase significantly at véraison [45]. External applications of ABA to ripening fruit can accelerate berry development (see [13] and references therein). The transcript abundance of 9-cis-epoxycarotenoid dioxygenase (NCED), which encodes the rate limiting step in ABA biosynthesis, increased during the lag phase and peaked at stage 35 around the start of véraison (Figure 5A). Both NCED1 (1608022_at, TC57089) and NCED4 (1607029_at, TC55541) had similar expression patterns, but differed significantly in their relative trancript abundance. A transcript (1614892_at, TC54474) encoding ABI1 (protein phosphatase 2C) showed an expression pattern like that of the NCED genes, but was more highly correlated with NCED4 than NCED1. The RD22 gene (1619802_at, TC67323), a dehydration-responsive protein, displayed a very large increase in abundance at véraison that continued to increase during berry maturation, whereas another transcript (1621346_at, TC65114) encoding an ABI3/VP1 (ABscisic acid Insensitive 3/ViviParous 1) transcription factor showed highest transcript abundance during the lag phase.

### Ethylene

Traditionally, wine grape has been considered a non-climacteric fruit, however, there are studies that indicate that ethylene plays an important role in berry development and ripening [13] and is required for increased berry diameter and ripening processes, such as anthocyanin biosynthetic gene expression and accumulation [46,47]. In addition, ethylene appears to be involved in controlling the expression of an alcohol dehydrogenase gene from grape [48]. Furthermore, some inhibitors of ethylene biosynthesis can delay berry ripening [49]. Ethylene-related transcripts displayed some very unique and intriguing patterns of expression (Figure 5B) indicating that this signaling pathway is differentially expressed along berry development. One transcript (1617012_at, TC68057) encoding a putative Ethylene Response Factor 1 (ERF1), a putative ethylene output gene, displayed a steady increase in abundance with maximal expression at ripening (Figure 5B) indicating a potential post-véraison role for this signaling pathway. An ethylene-induced transcription factor (1619585_at, TC62897) exhibited transcript accumulation during the lag (E-L stages 32 to 34) and early véraison (E-L stage 35) stages of development. A putative ethylene co-activator (1621552_at, TC66829) protein displayed biphasic peak transcript abundance at E-L stages 32 and 35. The transcript abundance of ACC oxidase (1615952_s_at, TC56709), the enzyme responsible for the last step in ethylene biosynthesis, was highest at E-L stage 32, the start of the lag phase, and then declined throughout the remainder of berry development. Interestingly, the transcript abundance of an ethylene receptor ERS1 (1622402_at, TC62349) and EIN4/ETR5 (1618518_at, TC55908) were at their lowest during E-L stages 32 to 33 until véraison, but then increased at a later stage (E-L stage 38). Ethylene pathway activation in grape berry appears to occur within a three week period of berry development (weeks 6 to 8 after anthesis; E-L stages 30 to 33) when the highest ethylene (ACC) content and transcript abundance of ACC oxidase were detected in Cabernet Sauvignon [46]. This hypothesis is supported by the observation that application of exogenous ethylene 8 weeks after anthesis hastened the ripening of the grape berries and resulted in a decrease in average cell size. In contrast, if the same ethylene treatment was applied during earlier stages of berry development (at 4, 5, 6 or 7 weeks), maturation was delayed [47].

According to the *Arabidopsis *model of ethylene signaling, reduced expression of transcripts and activity of receptors increases the sensitivity to ethylene, whereas increased receptor expression and activity decreases sensitivity [50]. In tomato, the expression of most genes encoding ethylene receptors increases during fruit development. In parallel, high levels of ethylene are expressed to counterbalance the negative effect of increased receptor expression on the ethylene signaling pathway [51]. In grape berry, the slight decreases observed in ethylene receptor transcript expression occurring between E-L stages 31 and 32 and the peak of ethylene accumulation during this same period, indicate a higher sensitivity to ethylene during the early stages of berry development. This would be expected to lead to a greater activation of the ethylene signaling pathway prior to véraison.

As in grape berry, strawberry is able to produce significant levels of ethylene during fruit development, but not to the same extent as climacteric fruits. Recently, three ethylene receptors have been identified in strawberry [52]. Two of them (FaEtr1 and FaErs1) display the same pattern of expression during fruit development as those observed for ERS1 ethylene receptor during grape berry development. In addition, the highest rates of ethylene production in strawberry were detected in very young green fruits. Following this, the hormone decreases continuously until the White stage of fruits. Following this stage, ethylene showed a slight but steady increase for the remainder of development. When considered together, the similarities of expression of ethylene receptors during fruit development for both grapes and strawberries coupled with the concomitant ethylene production during the early steps of fruit development indicate a conserved mechanism for ethylene perception between these fruits prior to ripening.

### Brassinosteroids

Brassinosteroids (BR) have recently been implicated in playing an important role in berry development [53]. Castasterone concentrations are low during the early stages of berry development and then increase at véraison [53]. Brassinosteroids have been shown to increase cell size [54] indicating that berry enlargement may be affected by castasterone levels. BRH1 RING finger protein (1617572_at, TC66046) transcript abundance, which is known to be down-regulated by exogenous application of BR, decreased during E-L stages 31 to 35, but increased in fully mature berries (Figure 5C). The transcript abundance of the Brassinosteroid Receptor 1 gene (BRI1, 1612516_at, TC56501) peaks at the start of the lag phase (E-L stage 32) and then declines thereafter. The transcript abundance of BRU1 (1608945_at, TC54729), which is a BR-responsive transcript encoding a xyloglucan endotransglycosylase (XET), showed a transient increase in abundance at véraison. In the same family, transcripts for another BR-responsive protein (1619068_at, TC60314) declined with berry development. Clearly, there are many significant changes in transcript abundance that are associated with brassinosteroid responses during berry development.

### Gibberellins

Very little is known about the role of gibberellin (GA) in grape berry development except a possible role in cell enlargement. Biologically active concentrations of GA are high in flowers and in fruits just after anthesis, but then drop to lower levels over the course of berry development [53,55]. There is a second peak of active GA at the start of the lag phase and it is 77 times higher in the seed compared to the berry mesocarp [56]. The transcript abundance of two putative GA receptors, GIDL1 and GIDL2 (1618181_at, TC67464; 1620071_at, TC56624, respectively), increased during berry development (Figure 5D). Interestingly, the transcript abundance of the GA signaling pathway repressor, GAI1 (1606777_s_at, TC56894), declines transiently at véraison. The transcript abundance of a putative GA β-hydroxylase (1610610_at, TC66284) declines over the course of berry development (Figure 5D) more or less coincident with the known accumulation pattern of GA_1 _in developing berries.

### Auxins

The mechanisms by which the phytohormone indole-3-acetic acid (IAA) regulates berry development are complex and not fully understood. Increased auxin production produced through the action of an ovule-specific auxin-synthesizing transgene enhanced fecundity in grapes [57]. Earlier reports indicated that auxin concentrations were high during early Phase I and declined following véraison [55] consistent with the role of this phytohormone in promoting cell division and expansion during Phase I. Treatment of grape berries with synthetic auxin-like compound, benzothiazole-2-oxyacetic acid (BTOA) delayed ripening [45]. A more recent study showed that auxin concentrations remain relatively constant over the course of berry development [53].

Our data indicate that there are numerous transcript responses to auxin (Figure 5E). The transcript abundance of Aux22 (1614660_at, TC53887), which forms heterodimers with auxin response factors (ARF) in order to repress auxin responses, increased after véraison (Figure 5E). Transcripts for both Auxin Response Factor 2 (ARF2, 1613813_a_at, TC65541) and a Small Auxin Up RNA protein (SAUR) (1609591_at, TC63193) increased after véraison, whereas transcripts for a different SAUR transcript (1606566_at, TC62299) and an Auxin-induced Response Factor, ARF18 (1616225_at, TC52772) both declined in a very similar pattern during berry development. A transcript (1619610_at, TC56575) encoding IAA-amino acid hydrolase, which is involved in IAA homeostasis, was highly expressed during the later stages of berry development (Figure 5E). The synthesis and hydrolysis of IAA conjugates, which function in both permanent inactivation and temporary storage of auxin [58], may play an important role in the control of IAA concentrations as berry development progresses. IAA-amino acid hydrolase may provide for local concentrations of auxins within the berries to promote mesocarp cell enlargement. Several transcripts (1611479_at, CD799903; 1617179_at, CF414958; 1610034_at, TC59892) related to auxin transport and perception also displayed increased abundance at the onset of véraison. Given the importance of auxin-mediated processes in developing berries, more research needs to be conducted to elucidate the mode of action of auxin signaling and response pathways.

### Methyl jasmonate and cytokinins

Methyl jasmonate (MeJA) is known to promote the synthesis and accumulation of terpenes and resveratrol in berry cell cultures [59,60], however, its effects *in vivo *are not well understood. The transcript abundance of 12-oxophytodienoate reductase (12-OPR) (1607601_at, TC61395), which is involved in jasmonate biosynthesis [61], and a constitutive pathogen-response 5 protein (1614324_at, CF213899), both decreased with berry development (Figure 5F). Less is known about the role of cytokinins in berry development. The transcript abundance of cytokinin oxidase (1620306_at, TC69712), which degrades cytokinin [62], decreased over berry development, whereas a known cytokinin-response regulator, a Type-A response regulator (1612955_at, TC52530), showed a steady increase in transcript abundance over berry development (Figure 5F).

### New candidates genes associated with calcium signaling, flavonoid transport and flavor

**Figure 6:**
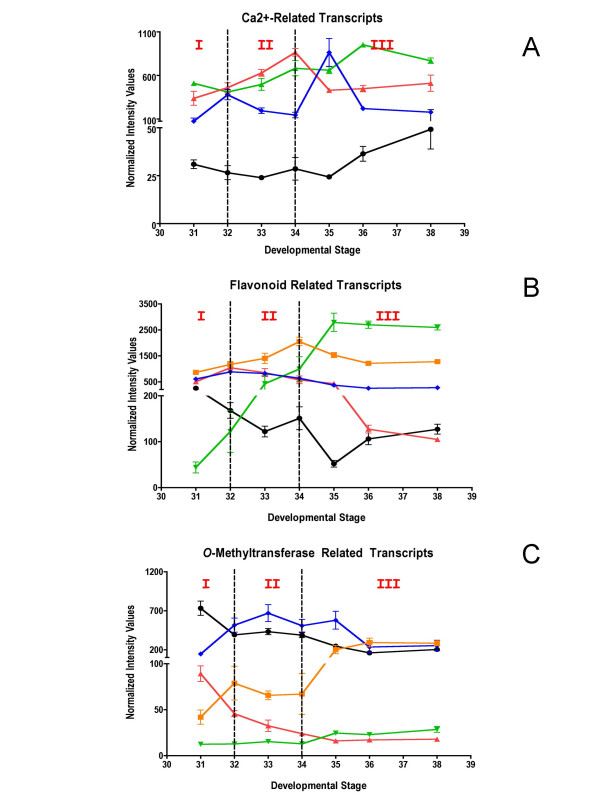
**Expression of potential candidates Unigenes**. **A) **Black solid round (1614028_at, TC67285)-cation-transporting ATPase, red solid triangle (1622073_at, CF404214)-calcium-transporting ATPase, green solid triangle (1617237_s_at, TC66680)-Ca^2+^/H^+ ^exchanger, blue solid diamond (1618587_at, TC64370)-calmodulin-repressor of gene silencing. **B) **Black solid round (1619917_s_at, TC69505)-glutathione-*S*-transferase, red solid triangle (1609870_at, TC58286)-glutathione-S-transferase conjugating ATPase, green solid triangle (1607560_at, TC62162)-multi-drug secondary transporter like protein (MATE), blue solid diamond (1611091_s_at, TC54724)-VvMYBPA1, orange solid square (1618504_at, TC61713)-MYC transcription factor. **C) **Black solid round (1608603_at, TC56956)-phloroglucinol O-methyltransferase, red solid triangle (1613542_at, TC62584) O-methyltransferase, green solid triangle (1620469_at, CF209780)-O-methyltransferase, blue solid diamond (1616348_at, TC52353)-S-adenosyl-L-methionine:benzoic acid/salicylic acid carboxyl methyltransferase orange solid square (1612552_at, TC57170)-S-adenosyl-L-methionine:salicylic acid carboxyl methyltransferase.

Calcium has many essential roles in plant growth and development [63], however, the role of calcium signaling in grape berry development is largely unexplored. Recently, an ABA-responsive calcium-dependent protein kinase (CDPK) was described that was specifically expressed in the seed and flesh of berries with increased transcript abundance over berry development and ripening [64]. In the current study, a large number of genes with functions related to calcium sequestration, transport and signaling were found to display developmentally regulated expression patterns (Figure 6A; Table 3).

**Table 3:** Transcripts (TFR pool) related to calcium categorized by the first hit in the MIPS2 catalog

**Probeset ID**	**GenBank Annotation**	**VvGI5**	**UniProt ID**	**Gene Name Description**	**Function**	**Profile**	**Fold Change**
1616662_at	CF404703	TC59643	Q9LIK7	Ca2+/ATPase	Ca transport	3	27.96
1617237_s_at	CF207946	TC66680	O64455	Ca2+/H+ exchanger (VCAX1)	Ca transport	14	2.56
1614028_at	CB976052	TC62785	Q7X8B5	Ca2+-transporting ATPase 8	Ca transport	16	2.32
1619731_at	CB972437	CB972437	Q93YX7	Type IIB calcium ATPase	Ca transport	21	2.2
1622073_at	CF404214	CF404214	Q9LIK7	Calcium-transporting ATPase 13	Ca transport	5	2.05
1615486_at	CF415476	TC69351	Q5D6H2	Cyclic Nucleotide-Gate Channel 2	Ion channel	3	8.21
1621591_at	CB981532	TC66482	Q94AS9	Cyclic nucleotide-gated ion channel 4	Ion channel	10	3.01
1609527_at	CD802146	TC64117	Q6ZHE3	Cyclic nucleotide-binding transporter 1	Ion channel	8	2.06
1613268_at	CB342482	TC53213	O65717	Cyclic nucleotide-gated ion channel 1	Ion channel	12	2.05
1614456_at	BQ797488	BQ797488	Q8L706	Ca2+-dependent lipid-binding protein	Lipid binding	11	3.83
1614582_at	BQ799084	BQ799084	Q8LJ85	Calreticulin	Protein folding and stabilization	12	2.37
1611917_at	CB972164	TC58290	Q39817	Calnexin	Protein folding and stabilization	1	2.31
1612291_at	CB347450	TC67746	P93508	Calcium-binding protein	Protein folding and stabilization	3	2.2
1622324_at	CF568845	TC63952	Q39817	Calnexin	Protein folding and stabilization	1	2.04
1612443_at	CF211151	TC68392	Q7X996	CBL-interacting protein kinase 20	Signal transduction	11	12.97
1610295_at	BQ797947	TC57947	Q8W1D5	CBL-interacting protein kinase 5	Signal transduction	4	12.35
1618587_at	CF518131	TC64370	Q9AXG2	Calmodulin	Signal transduction	21	11.43
1618447_at	CA815141	TC53225	Q6ETM9	CBL-interacting protein kinase 21	Signal transduction	3	7.75
1611127_at	CF510878	TC64442	Q8L3R2	Calmodulin	Signal transduction	12	5.33
1610922_at	CF404315	TC68116	Q1SFZ7	CBL-interacting protein kinase 21	Signal transduction	3	3.43
1606980_at	CF211606	TC69501	Q008R9	Calcium sensor homolog	Signal transduction	2	3.23
1618045_at	CF216119	TC53057	Q676U1	CBL-interacting protein kinase 20	Signal transduction	21	2.9
1611172_at	CB003645	TC52484	Q8LK24	SOS2-like protein kinase	Signal transduction	16	2.81
1612269_at	CB345885	TC53895	Q3HRN8	Calcineurin B	Signal transduction	13	2.74
1606859_at	CF518881	CF518881	Q3HRN8	Calcineurin B	Signal transduction	13	2.74
1613576_s_at	CF201676	TC60874	P62200	Calmodulin 1/11/16	Signal transduction	11	2.7
1622351_at	CA810859	TC60874	P62200	Calmodulin 1/11/16	Signal transduction	11	2.26
1611555_at	CB971903	TC54154	Q9SS31	Calmodulin-related protein 2	Signal transduction	13	2.22
1608587_at	CD799705	TC62151	Q5D875	Calcium-dependent protein kinase CDPK1444	Signal transduction	10	2
1614600_s_at	CF213754	TC52150	Q9ZT86	Calcium-binding protein	Unclassified protein	7	2.89
1616580_at	CF206767	TC55591	Q84Y18	CAX-interacting protein 4	Unclassified protein	11	2.63

Calcium homeostasis within the cytosol is tightly controlled by membrane spanning Ca^2+^-ATPases and H^+^/Ca^2+ ^exchangers, which typically maintain low concentrations of Ca^2+ ^in the cytosol and restore this concentration following signaling-related transient changes in calcium levels. Transcripts encoding plasma membrane Ca^2+^-ATPase genes (1614028_at, TC62785; 1622073_at, CF404214), which are closely related to ACA8 and ACA13, respectively, in *Arabidopsis thaliana*, showed increased transcript abundance during E-L stages 33 and 34 and in later developmental stages. Interestingly, ABA markedly and rapidly stimulates the expression of the *ACA8 *gene in cell cultures of *Arabidopsis thaliana *[65]. A tonoplast Ca^2+^/H^+ ^exchanger (1617237_s_at, TC66680), which is a close homolog of CAX3 from *A. thaliana *and plays a key role in cytosolic Ca^2+ ^homeostasis [66], showed a transient increase in transcript abundance at E-L stages 34, indicating a possible role for calcium signaling around véraison.

ABA accumulates until two weeks after the beginning of véraison before decreasing later in berry development [67]. Thus, it is likely that ABA is directly or indirectly involved in the control of Ca^2+ ^signaling and homeostasis events, particularly around véraison.

The increased expression of several Unigenes encoding calmodulin or calcium interacting protein kinases (see Table 3) supports this hypothesis [68]. One Unigene encoding a calmodulin-related suppressor of gene silencing (1618587_at, TC64370) displayed a pronounced pattern with two peaks of expression at E-L stage 32 and at E-L stage 35 corresponding to two transitions of berry development (Phases I to II and Phases II to III). This Unigene displayed a 10-fold change in its transcript abundance across berry development and may be involved in the suppression of posttranscriptional gene silencing (PTGS) by interacting with a proteinase known to suppress PTGS in plants [69]. This correlation indicates a possible role for calcium in regulating the activity of the PTGS mechanisms. To date, only one paper reported the possibility of the involvement of PTGS in the regulation of gene expression during plant development [70]. Further investigations are necessary to evaluate the real impact of this Unigene in the triggering of véraison.

Phenolic compounds, derived from flavonoids (anthocyanins, tannins and flavonols), are the major wine constituents responsible for organoleptic properties such as color and astringency. Twenty-one Unigenes encoding biosynthetic enzymes of the general phenylpropanoid and flavonoid pathways were found to exhibit differential mRNA expression patterns across berry development (Table 4). The vast majority of these genes are expressed predominantly in the skin [71].

**Table 4:** Transcripts (TFR pool) related to flavonoid metabolism categorized by the first hit in the MIPS2 catalog within specific sub-sections of the flavonoid pathway

**Probeset ID**	**GenBank Annotation**	**VvGI5**	**UniProt ID**	**Gene Name Description**	**Function**	**Profile**	**Fold Change**
1617171_s_at	AF000371	TC51696	O22303	UDP glucose:flavonoid 3-o-glucosyltransferase (UFGT)	Anthocyanin Pathway	11	46.79
1614441_at	BQ798241	TC57653	Q9SWY6	Anthocyanidin synthase (ANS)	Anthocyanin Pathway	11	12
1618112_at	CB971725	TC70789	Q9LTA3	Anthocyanidin-3-glucoside rhamnosyltransferase	Anthocyanin Pathway	3	9.39
1611309_at	CF210457	TC58629	Q8H1R1	Dihydroflavonol 4-reductase (DFR)	Common Pathway	19	7.36
1611739_at	CF403783	TC64266	Q8H224	Flavonoid 3'-hydroxylase (F3'H)	Common Pathway	2	5.68
1620675_at	CB969894	TC51699	P93799	Dihydroflavonol 4-reductase (DFR)	Common Pathway	3	5.21
1617019_at	BQ800456	TC67173	O80407	Chalcone synthase (CS)	Common Pathway	3	5.17
1607739_at	CF415693	TC70298	P41090	Flavanone 3-hydroxylase (F3H)	Common Pathway	3	2.93
1608379_at	CF202029	TC40489	Q8H8H7	Flavanone 3-hydroxylase (F3H)	Common Pathway	21	2.55
1607732_at	AF020709	TC63806	O22519	Chalcone synthase (CS)	Common Pathway	3	2.48
1608761_at	CB982029	TC53331	Q9FLV0	Flavanone 3-hydroxylase (F3H)	Common Pathway	18	2.02
1611542_at	CB971080	TC51691	P43311	Polyphenol oxidase (PPO)	Flavonoid Catabolism	3	28.9
1622651_at	CF215945	TC58764	P93622	Polyphenol oxidase (PPO)	Flavonoid Catabolism	5	3.79
1608791_at	CB978059	TC66577	Q84TM1	Flavonol synthase (FLS5)	Flavonol Pathway	3	5.12
1621051_at	CN006197	-	Q40285	Flavonol 3-O-glucosyltransferase	Flavonol Pathway	13	3.94
1615401_at	CB342555	TC55331	Q40285	Flavonol 3-O-glucosyltransferase	Flavonol Pathway	15	2.43
1618155_at	CD004374	TC54048	Q40288	Flavonol 3-O-glucosyltransferase 6	Flavonol Pathway	10	2.27
1612134_at	CF204393	TC53206	Q5FB34	Anthocyanin reductase (ANR)	Proanthocyanidin Pathway	3	34.12
1615174_s_at	CD011073	TC68741	Q4W2K6	Leucoanthocyanidin reductase 2 (LAR2)	Proanthocyanidin Pathway	13	4.08
1608212_at	CK138122	TC54322	Q84V83	Leucoanthocyanidin reductase 2 (LAR2)	Proanthocyanidin Pathway	13	3.52

The mechanisms by which anthocyanins accumulate in the vacuole of grape berry skin cells during Phase III are not fully understood. These compounds must be transported from the site of synthesis in the cytosol to their final destination, the vacuole. Several models have been proposed for sequestering anthocyanins in the vacuole in *Arabidopsis thaliana*. One model [72] indicates the action of a glutathione-S-transferase (GST) in facilitating the transfer of anthocyanins into the vacuole. Another model indicates that a transporter of the multidrug-resistance-associated protein family could facilitate the transport of an anthocyanin-GST complex into the vacuole [73]. Here, the Unigene transcript encoding a GST (1619917_s_at, TC69505; Figure 6B) displayed a 63-fold increase in abundance during the stages in berry development in which flavonoids accumulate (Figure 6B). This Unigene is closely related to a GST homolog known to be involved in anthocyanin sequestration [74]. This Unigene also displays a skin-specific expression pattern [71], which is consistent with the tissue localization of anthocyanins. A Unigene homologous to a glutathione-S-conjugate transporting ATPase (1609870_at, TC58286) showed a peak of expression at véraison (E-L stage 34). While not yet characterized in detail, this Unigene belongs to the ABC transporter sub-family, members of which are known to transport anthocyanins [74]. The putative multi-drug transporter (1607560_at, TC62162), which is known to be involved in the sequestration of tannins into vacuoles [75], exhibited peak transcript abundance at E-L stage 32 followed by a decline, and is consistent with the pattern of maximal tannin accumulation that occurs a few weeks before véraison.

Specific members of the MYB transcription factor family play critical roles in the regulation of flavonoid metabolism during grape berry development [76]. We detected four transcripts encoding MYB transcription factors that have been previously characterized in grape berry (see Table 4) [77-80]. VvMYBPA1 (1611091_s_at, TC54724) regulates the proanthocyanidin (condensed tannins) pathway in the grape berry [77]. In the Shiraz cultivar, VvMYBPA1 peak expression appears to occur during E-L stages 34 and 35 in the skin and seeds, whereas, in Cabernet Sauvignon this gene is expressed at an earlier developmental stage (E-L stages 32) (Figure 6B). Such differences are likely to be cultivar-dependent. In the same way, the MYC family of transcription factors also plays a key role in regulation of the anthocyanin pathway. One MYC transcription factor transcript (1618504_at, TC61713), which shares strong amino acid sequence identity with MYC genes known to be involved in the regulation of anthocyanin production [81], displayed a pattern of transcript accumulation that decreased from the beginning of berry development until E-L stage 35 and then increased for the remainder of fruit development (Figure 6B). Furthermore, this Unigene is preferentially expressed in the skin [71]. These expression patterns correlate well with the accumulation of anthocyanins and proanthocyanins.

In grape berries, volatile aroma compounds, such as terpenes, benzenoids, and phenylpropanoids, accumulate in exocarp and mesocarp tissues following the initiation of berry ripening [38,82]. Three transcripts (Figure 6C) encoding O-methyltransferases, which may participate in the biosynthesis of volatile compounds, were also detected [83]. The first Unigene (1608603_at, TC56956), which encodes a putative phloroglucinol O-methyltransferase, is involved in the biosynthesis of volatile 1,3,5-trimethoxybenzene, a compound not previously described in grape [83], displayed a very high transcript abundance at the beginning of berry development (E-L stage 31) before decreasing after véraison until E-L stage 36 and then increasing again in mature berries (Figure 6C). The second Unigene (1613542_at, TC62584) was expressed at E-L stage 31, but then declined. The third Unigene (1620469_at, CF209780) displayed very low transcript abundance with a slight increase following véraison (Figure 6C). Finally, two S-adenosyl-L-methionine (SAM):salicylic acid carboxyl methyltransferases were identified with developmentally-induced expression patterns. The first Unigene (1616348_at, TC52353) showed a broad peak of expression between E-L stages 32 to 35, whereas the second Unigene (1612552_at, TC57170) showed increased transcript abundance after véraison (E-L stage 34) (Figure 6C). Such genes are thought to play important roles in scent production or plant defense [84]. Little correlation between the level of sequence similarity and the structural similarity of their substrates has been observed for most of these protein families, so that gene functions have to be assigned following detailed biochemical testing [85].

### Organic acid and proline metabolism

**Figure 7:**
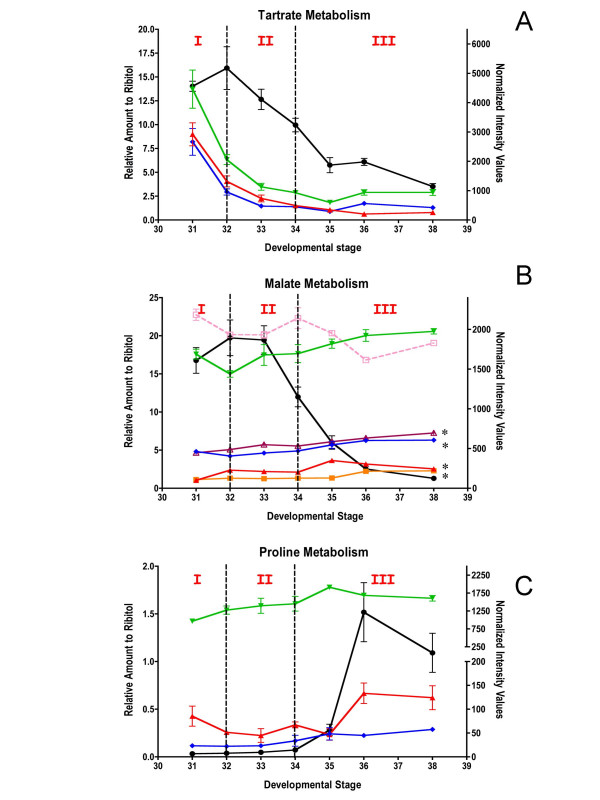
**Organic acids and amino acids: metabolites and transcripts**. **A) **Black solid round-tartrate, red solid triangle (1622252_at, TC52651)-L-idonate dehydrogenase, green solid triangle (1613165_s_at, TC52651)-L-idonate dehydrogenase, blue solid diamond (1612918_at, TC52651)-L-idonate dehydrogenase. **B) **Black solid round-malate, red solid triangle (1612546_at, TC68207)-cytosolic MDH, green solid triangle (1609147_at, TC55437)-cytosolic MDH, blue solid diamond (1622059_at, TC69439)-mitochondrial malate dehydrogenase (MDH), orange solid square (1617448_at, TC54982)-mitochondrial MDH, lavender open square (1609345_s_at, TC57092)-malic enzyme. **C) **Black solid round-proline, red solid triangle (1619565_at, TC52705)-pyrroline-5-carboxylate synthetase, green solid triangle (1617293_s_at, BQ792635)-proline dehydrogenase, blue solid diamond (1610800_at, CK906448)-proline transporter. *: Transcripts that do not pass the two-fold ratio. All compounds amounts were normalized by a ribitol standard (25 mg/L).

The acid:sugar balance at harvest is an important factor of wine quality as it affects important sensory attributes [15]. Two major organic acids that contribute to titratable acidity, tartrate and malate, are the most abundant organic acids in grapes and reach maximal concentrations around the end of Phase I (E-L stage 32; see Table 5). Tartrate concentrations were found to peak at E-L stage 32 and then declined steadily until harvest, E-L stage 38 (Figure 7A). Tartrate concentrations decreased in parallel with three different transcripts encoding L-idonate dehydrogenase (1622252_at, TC52651; 1613165_s_at, TC52651; 1612918_at, TC52651), a key enzyme in tartrate biosynthesis [86]. The innermost region of the berry pulp surrounding the seed has been shown to contain the highest tartrate concentrations [87]. Consistent with this observation, tartrate synthase transcripts have been shown to be more abundant in seeds than in outer mesocarp and skin tissues [71].

**Table 5:** Transcripts (TFR pool) related to organic and phenolic acid metabolism

**Probeset ID**	**GenBank Annotation**	**VvGI5**	**UniProt ID**	**Gene Name Description**	**Function**	**Profile**	**Fold Change**
1608526_at	CB974198	TC66314	Q5NBP4	AOBP-like protein	Organic Acid	11	6.48
1618209_at	CF373021	TC56127	P82281	Ascorbate peroxidase	Organic acid	3	3.59
1617448_at	BQ795936	TC54982	Q9M6B3	Malate dehydrogenase	Organic Acid	10	3.48
1606935_at	CB969531	TC66898	Q9SAK4	Succinic semialdehyde dehydrogenase 1	Organic acid	3	3.05
1620641_at	CF511421	TC52472	Q39540	AOBP-like protein	Organic Acid	9	2.11
1611871_at	CF415063	TC54132	Q84UH4	Dehydroascorbate reductase	Organic Acid	19	2.1
1609147_at	CB979150	TC55437	Q645N0	Malate dehydrogenase (cytosolic)	Organic Acid	11	2.01
1607417_at	CF512464	TC53733	Q8L7U8	Cinnamyl-alcohol dehydrogenase CAD1	Phenolic Acid	2	80.03
1614643_at	CF214966	TC51729	Q43237	Caffeoyl-CoA O-Methyltransferase	Phenolic Acid	2	34.34
1611265_at	CF513719	TC51900	Q49LX7	4-coumarate:CoA ligase	Phenolic Acid	11	25.11
1620342_at	CF207053	TC64352	Q00763	Caffeic acid 3-O-methyltransferase 1	Phenolic Acid	11	18.69
1610935_at	CF404728	TC64481	Q75W19	Ferulate-5-hydroxylase (FAH1)	Phenolic Acid	2	13.64
1619682_x_at	CF205002	TC62835	Q9M560	Caffeic acid O-Methyltransferase	Phenolic Acid	2	13.58
1616434_s_at	AF239740	TC62835	Q9M560	O-methyltransferase	Phenolic Acid	2	11.53
1609307_at	CD715818	TC66040	O24145	4-coumarate--CoA ligase (At4CL1)	Phenolic Acid	2	10.6
1619450_s_at	CF215109	TC52364	Q00763	O-methyltransferase	Phenolic Acid	2	10.28
1607475_s_at	CD012393	TC64352	Q3SCM5	Caffeic acid O-methyltransferase	Phenolic Acid	11	9.32
1614423_at	CF517687	TC66815	Q6DMZ8	Cinnamoyl CoA Reductase	Phenolic Acid	2	8.61
1620650_s_at	CF207485	TC69704	Q9ATW1	Cinnamyl-alcohol dehydrogenase	Phenolic Acid	1	5.95
1616191_s_at	CB971061	TC70715	Q3HM04	Cinnamate-4-Hydroxylase	Phenolic Acid	3	5.78
1613542_at	CF209028	TC62584	Q7X9J0	O-methyltransferase	Phenolic Acid	2	5.54
1622267_at	CF516149	TC64537	O65152	Cinnamyl-alcohol dehydrogenase	Phenolic Acid	3	4.48
1619320_at	CB974305	TC66743	P31687	4-coumarate:CoA ligase 3 (4CL3)	Phenolic Acid	3	4.21
1619808_at	CB972340	TC54722	O65152	Cinnamyl-alcohol dehydrogenase	Phenolic Acid	3	3.96
1611249_s_at	CF517155	TC51769	O65152	Cinnamyl-alcohol dehydrogenase	Phenolic Acid	3	3.93
1613831_at	CD801016	TC58955	Q5I6D6	Sinapyl alcohol dehydrogenase	Phenolic Acid	3	3.36
1613548_at	CB009193	TC68990	Q8H8C9	4-coumarate:CoA ligase	Phenolic Acid	11	3.19
1615439_at	CF213244	TC63112	P30359	Cinnamyl alcohol dehydrogenase 2	Phenolic Acid	2	2.38
1609327_at	CF208599	TC68572	A1YIQ2	Cinnamyl-alcohol dehydrogenase 1	Phenolic Acid	2	2.33
1607163_at	CF415171	-	Q8LSQ3	4-coumarate:CoA ligase	Phenolic Acid	3	2.22
1613511_at	BQ796246	TC59682	Q65CJ7	Hydroxyphenylpyruvate reductase	Phenolic Acid	11	2.22
1616445_at	CD716014	TC57545	Q9LYJ0	Cinnamoyl CoA Reductase	Phenolic Acid	16	2.11

Like tartrate, malate concentrations peaked around E-L stage 32, but then declined more rapidly than tartrate during berry ripening (Figure 7B). In contrast to the good correlation between tartrate and L-idonate dehydrogenase transcript abundance, there is a less obvious correlation between malate concentrations and the transcript abundance of Unigenes encoding malate dehydrogenases (Figure 7B). Transcript abundance for two isogenes encoding cytosolic NAD-dependent malate dehydrogenases (1612546_at, TC68207; 1609147_at, TC55437), which catalyze the interconversion of malate to oxaloacetate, increased during ripening. Transcripts for mitochondrial isoforms of the enzyme (1622059_at, TC60439; 1617448_at, TC54982) also increased over this same time period. In contrast, the transcript abundance of a NADP-dependent malic enzyme (1609345_s_at, TC57092), which catalyzes the oxidative decarboxylation of malate to pyruvate, declined slightly from E-L stages 34 to 36, but then increased by stage 38 (Figure 7B). The slight increase in the expression of all of these enzymes together may contribute to the declining concentrations of malate during ripening. Very little is known about the mechanisms of malate transport processes in the phloem/xylem and within developing grape berries. The regulation of malate concentrations in berries appears to be quite complex. More research is needed to elucidate this well known developmental process.

Mature berries contain unusually high concentrations of free proline; proline being the most abundant amino acid in Cabernet Sauvignon [88,89]. Proline concentrations increased significantly at véraison and remained high until berries were fully ripe (Figure 7C). Transcripts encoding pyrroline-5-carboxylate synthetase (1619565_at, TC52705), the key regulatory enzyme in proline biosynthesis, remained relatively constant with a small peak of expression occurring at E-L stage 35 (Figure 7C). Proline dehydrogenase transcripts (1617293_s_at, BQ792635), which encode the first enzymatic step in proline catabolism, increased only during the latter stages of berry development. These mRNA expression patterns are consistent with earlier reports and with protein expression patterns of these enzymes [88]. Proline accumulation correlated poorly with steady-state transcript and protein abundance changes for these two enzymes indicating that proline production is regulated by posttranslational mechanisms [88]. Steady-state transcripts encoding a proline transport protein (1610800_at, CK906448) also increased in conjunction with proline abundance.

### Sugar metabolism

Sugar accumulation in grape berries has been well studied because sugar content is a key factor in producing wine. In contrast to organic acids, hexose sugars (i.e., Glc and Fru) begin to accumulate substantially in the lag phase (Phase II) and continue thereafter. In grapevines, carbohydrates produced during photosynthesis are exported from the leaf as sucrose and transported in the phloem to the berry cluster [90,91]. Prior to véraison, most sugars imported into the berries are metabolized with little if any storage of these compounds. Following véraison, however, sugars accumulate in the vacuole to high levels in the form of glucose and fructose following the enzymatic cleavage of sucrose (mainly in the apoplast, but also in the cytoplasm and vacuole). Monosaccharide transporters direct the transport of these sugars through different organelles [92].

**Figure 8:**
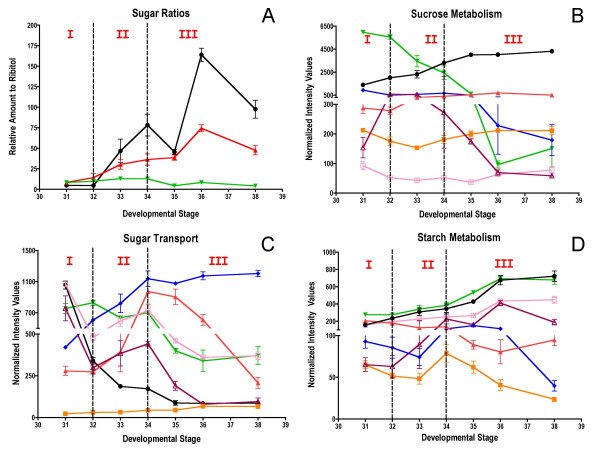
**Hexose sugars, transporters, and starch: metabolites and transcripts**. **A) **Black solid round-fructose, red solid triangle-glucose, green solid triangle-sucrose. **B) **Black solid round (1609402_at, TC62599)-sucrose synthase, red solid triangle (1608257_at, TC68135)-sucrose-6-phosphate phosphatase, green solid triangle (1611613_at, TC60693)-invertase (GIN1), blue solid diamond (1612836_at, TC57719)-invertase (GIN2), orange solid square (1620628_at, TC67908)-neutral invertase, lavender open square (1611027_at, TC56057)-acidic invertase, brown open triangle (1616255_at, TC57339)-fructokinase. **C) **Black solid round (1616083_at, TC51694)-VvHT1 (hexose transporter 1), red solid triangle (1615257_at, TC65400)-VvHT6 (hexose transporter 6), green solid triangle (1615697_at, TC51724)-VvSUC27 (sucrose transporter), blue solid diamond (1608991_at, TC60060)-plastidial glucose transporter, orange solid square (1613408_at, TC66667)-polyol transporter, lavender open square (1619379_at, TC58801)-plastidial triose phosphate transporter, brown open triangle (1622157_at, TC61733)-plastidial triose phosphate transporter. **D) **Black solid round (1615571_at, TC53551)-starch synthase, red solid triangle (1613601_at, TC67353)-starch synthase, green solid triangle (1617068_at, TC54621)-plastidial alpha-glucan, water dikinase, blue solid diamond (1617941_at, TC62494)-plastidial alpha-glucan, water dikinase, orange solid square (1622120_at, TC54533)-starch phosphorylase, lavender open square (1613188_at, TC70258)-α-amylase, brown open triangle (1617124_at, TC67979)-β-amylase. All compounds amounts were normalized by a ribitol standard (25 mg/L).

In the berries in this study, fructose was more abundant than glucose; in contrast sucrose concentrations remained relatively low and constant throughout berry development (Figure 8A). Transcript abundance for the Unigene encoding sucrose synthase (1609402_at, TC62599), increased gradually over berry development consistent with increased hexose accumulation in the berry. This Unigene has high homology with the sucrose synthase (CiTSUSA) in *Citrus unshiu *[93]. CiTSUSA also increases with fruit development and catalyzes the reaction in the cleavage direction (sucrose to UDP-glucose and fructose). Komatsu et al. [93] suggest that the action of this gene may be important for sink strength.

Sucrose-6-phosphate phosphohydrolase (SPP) (1608257_at, TC68135), which catalyzes the last step in sucrose synthesis, showed a slight increase in transcript abundance after E-L stage 32 and then remained relatively constant throughout the remainder of berry development (Figure 8B). In contrast, the transcript abundance of two vacuolar invertases, GIN1 and GIN2 (1611613_at, TC60693; 1612836_at, TC57719), which catalyze the catabolism of sucrose to fructose and glucose, declined over the course of berry development (Figure 8B), consistent with an earlier report [94]. The mRNA expression of these two invertases is consistent with the early increases in sugar accumulation during Phase II (E-L stages 32 to 34). On the other hand, transcript abundance for a neutral invertase (1620628_at, TC67908) and a cell wall acid invertase (1611027_at, TC56057) remained relatively constant during the course of berry development consistent with earlier reports on the amount and activity of these enzymes in developing berries [95]. In grape berries, sucrose cleavage is largely catalyzed by cell wall bound invertases [95]. Sucrose cleavage is usually associated with cell wall invertase activity at the onset of ripening, together with a shift towards apoplastic phloem unloading of sugars in berries during this same period of time [95]. Finally, transcripts encoding fructokinase (1616255_at, TC57339), which catalyzes the formation of fructose-6-phosphate and may regulate starch formation, declined in abundance in a similar manner as GIN1 and GIN2 following a peak of expression at E-L stage 32.

In most sink cells, sucrose is either cleaved by invertase into glucose and fructose or degraded by sucrose synthase into uridine-5'-diphosphate (UDP) glucose and fructose for subsequent metabolism and biosynthesis [96,97]. Cell wall invertases appear to play the main role in the cleavage of sucrose during Phase III of berry development [95]. However, the increase in sucrose synthase during Phase III of berry development indicates that this isogene may participate in the catabolism of sucrose to fructose and glucose. Alternatively, this sucrose synthase isogene may play a critical role in cellulose synthesis associated with Phase III cell expansion similar to its role in cotton fiber elongation [98]. Two cellulose synthase isogenes (1607069_at, TC53461; 1611149_at, TC56091) displayed increased transcript abundance during Phase II and III, consistent with this hypothesis (see Table 1). Additional developmentally regulated transcripts related to carbohydrate metabolism and transport are summarized in Table 6.

**Table 6:** Transcripts (TFR pool) related to carbohydrate metabolism and transport categorized by the first hit in the MIPS2 catalog

Probeset ID	GenBank Annotation	VvGI5	UniProt ID	Gene Name Description	Function	Profile	Fold Change
1618061_a_at	CF514699	TC52548	O78327	Transketolase	Amino Acids Metabolism	20	3.44
1614105_at	CB968800	TC70460	Q4JIY3	Pyruvate dehydrogenase	Amino Acids Metabolism	11	2.2
1616700_at	CB910092	TC53526	Q9SLY2	Sucrose synthase	Carbohydrate metabolism	3	227.67
1611613_at	BQ796771	TC60693	Q9S944	Invertase	Carbohydrate metabolism	3	61.86
1622656_at	CF215745	TC61716	Q5NA70	Glucan endo-1,3-b-glucosidase	Carbohydrate metabolism	2	53.85
1614716_at	CB978853	TC58640	Q6Z8F4	Phosphoribulokinase	Carbohydrate metabolism	12	51.76
1617719_at	CB975632	TC55314	Q6IV07	UDP-glucose:protein transglucosylase	Carbohydrate metabolism	2	40.93
1607442_at	CF403717	-	Q50HW0	Glucuronosyltransferase	Carbohydrate metabolism	2	38.54
1622115_at	CD004218	TC60627	Q9SRX8	b-glucosidase	Carbohydrate metabolism	10	26.34
1611970_at	CF207195	TC62847	Q9LKY6	Glucose acyltransferase	Carbohydrate metabolism	3	25.74
1620679_at	CB972076	TC53351	Q9LV33	b-glucosidase	Carbohydrate metabolism	14	16.26
1621352_at	BQ794457	TC59789	Q8GT41	Invertase inhibitor	Carbohydrate metabolism	11	16.06
1608932_at	CB982469	TC63201	Q59J80	Glucosyltransferase	Carbohydrate metabolism	11	15.38
1620347_at	CA814065	TC66065	Q5QPZ6	Glycosyltransferase	Carbohydrate metabolism	10	15.36
1622282_at	CD712313	TC54393	Q7XAE2	Fructokinase	Carbohydrate metabolism	2	13.54
1616642_at	BQ800221	TC64250	Q9FEP9	Glycerol-3-phosphate acyltransferase	Carbohydrate metabolism	16	11.07
1616255_at	CF516475	TC57339	O82616	Fructokinase	Carbohydrate metabolism	12	10.13
1612918_at	CB972844	TC52651	Q9MBD7	NAD-dependent sorbitol dehydrogenase	Carbohydrate metabolism	2	9.14
1608393_at	CF403620	TC64860	O22658	ADP-glucose pyrophosphorylase	Carbohydrate metabolism	7	8.79
1621067_at	CF511425	TC51908	Q8W3C8	Glucose acyltransferase	Carbohydrate metabolism	3	8.2
1612883_at	CB911656	TC60606	O22060	Sucrose-phosphate synthase 1	Carbohydrate metabolism	16	7.92
1617035_s_at	CF205538	TC64995	Q9XGN4	Galactinol synthase	Carbohydrate metabolism	11	7.73
1609652_s_at	CF215703	TC59328	Q9FNI7	Glucosyltransferase	Carbohydrate metabolism	3	7.5
1617309_at	CB922444	TC59505	Q8LFT7	Aldehyde dehydrogenase	Carbohydrate metabolism	10	7.26
1619190_at	CD720196	TC54797	Q6H5W0	Alcohol dehydrogenase	Carbohydrate metabolism	3	6.66
1616107_s_at	CD715446	TC67979	Q94EU9	b-amylase	Carbohydrate metabolism	9	6.34
1618409_at	CF514784	TC52918	Q94G86	Glucan endo-1,3-b-glucosidase	Carbohydrate metabolism	3	6.26
1620624_at	CB969436	TC52478	Q94IP3	UDP-Glucose Transferase	Carbohydrate metabolism	2	6.21
1611680_at	CF415491	TC58448	Q50HU7	Glycosyltransferase	Carbohydrate metabolism	2	5.8
1612465_at	CF568806	TC53602	O65736	b-galactosidase	Carbohydrate metabolism	4	5.76
1618071_at	CF518536	TC54381	Q9M8Y0	O-linked GlcNAc transferase	Carbohydrate metabolism	16	5.7
1611804_at	CF513259	TC62252	Q9ZVX4	Glucose acyltransferase	Carbohydrate metabolism	12	5.52
1617454_at	BQ798893	-	Q8VYG2	Galactokinase	Carbohydrate metabolism	2	5.43
1612836_at	CF403299	TC57719	Q9S943	Invertase	Carbohydrate metabolism	3	5.28
1618517_at	CB971627	TC53602	Q93X58	b-galactosidase	Carbohydrate metabolism	4	5.01
1622074_at	BQ794083	-	Q84JP7	Phosphoenolpyruvate carboxylase kinase	Carbohydrate metabolism	12	4.66
1615571_at	CB983156	TC53551	Q9FNF2	Starch synthase	Carbohydrate metabolism	9	4.58
1622543_at	CB977855	TC61696	Q84V96	Aldehyde dehydrogenase	Carbohydrate metabolism	1	4.57
1610724_at	CB916342	TC63651	Q652S1	Fructose/tagatose bisphosphate aldolase	Carbohydrate metabolism	11	4.57
1620997_at	CD799067	TC63159	Q84LI1	Galactose dehydrogenase	Carbohydrate metabolism	2	4.55
1619223_s_at	CB005867	TC52910	Q9SLS2	Sucrose synthase	Carbohydrate metabolism	2	4.46
1615614_at	CF405918	TC54197	Q9M3I0	Glucosyltransferase	Carbohydrate metabolism	2	4.28
1622065_at	CD801714	-	Q94FA7	Fructose-bisphosphatase	Carbohydrate metabolism	3	4.17
1622503_at	CF203022	TC69704	Q9ATW1	Mannitol dehydrogenase	Carbohydrate metabolism	1	4.16
1615634_at	CB970085	TC69016	Q8L9U9	Glucose acyltransferase	Carbohydrate metabolism	12	3.86
1607324_at	CD719348	TC54773	P94078	a-mannosidase	Carbohydrate metabolism	2	3.85
1611112_at	CB971308	TC51885	Q7XPW5	Phosphomannomutase	Carbohydrate metabolism	3	3.85
1616325_at	CF211815	TC53040	Q6Q2Z9	Phosphoenolpyruvate carboxylase	Carbohydrate metabolism	3	3.84
1617068_at	CF519166	TC54621	Q9SGX4	Water dikinase	Carbohydrate metabolism	18	3.8
1611604_at	CB916873	TC54851	Q8LPJ3	a-mannosidase	Carbohydrate metabolism	3	3.78
1620724_at	CB915307	TC66445	O48628	Phosphofructo-1-kinase	Carbohydrate metabolism	11	3.73
1616500_at	AF194175	TC52882	Q9FZ00	Alcohol dehydrogenase	Carbohydrate metabolism	10	3.69
1612870_s_at	CF201540	TC66152	Q0DAH4	GDP-4-keto-6-deoxy-D-mannose-3,5-epimerase-4-reductase	Carbohydrate metabolism	3	3.66
1608527_at	CF515950	TC58983	Q9FJ95	Sorbitol dehydrogenase	Carbohydrate metabolism	10	3.6
1619457_at	CB969731	TC63406	P93653	Trehalose-6-phosphate synthase	Carbohydrate metabolism	12	3.58
1611154_at	CF204490	-	Q42954	Pyruvate kinase	Carbohydrate metabolism	3	3.54
1614552_at	CB978862	TC54160	Q5SMZ1	Aldose 1-epimerase	Carbohydrate metabolism	12	3.52
1608263_a_at	BQ794795	TC51761	Q9M6B4	Alcohol dehydrogenase	Carbohydrate metabolism	11	3.5
1617186_at	CF415580	TC70119	O65856	Glucose-6-phosphate dehydrogenase	Carbohydrate metabolism	1	3.49
1608907_s_at	CA809004	TC51713	Q9XGN4	Galactinol synthase	Carbohydrate metabolism	11	3.4
1613182_at	CB982869	-	Q6PP98	Pyruvate dehydrogenase kinase	Carbohydrate metabolism	11	3.37
1612414_at	CD715284	TC58601	Q42910	Pyruvate phosphate dikinase	Carbohydrate metabolism	10	3.34
1622120_at	CF519014	TC54533	P27598	Starch phosphorylase	Carbohydrate metabolism	21	3.33
1606536_at	CB971452	-	Q8S9A7	Glucosyltransferase	Carbohydrate metabolism	3	3.29
1622606_at	CB910226	TC52786	Q6DW08	GDP-mannose pyrophosphorylase	Carbohydrate metabolism	3	3.28
1610766_at	CF212685	TC53291	Q7Y152	Galactokinase	Carbohydrate metabolism	11	3.24
1615270_at	CF208284	TC70917	Q6K963	Callose synthase	Carbohydrate metabolism	21	3.23
1615167_at	CF519116	TC65652	Q9LFQ0	Glycosylation enzyme	Carbohydrate metabolism	12	3.2
1606774_at	CF415165	TC70261	Q8L7J4	Pyruvate kinase	Carbohydrate metabolism	11	3.14
1609402_at	BQ794844	TC62599	Q9SLY2	Sucrose synthase	Carbohydrate metabolism	11	3.09
1608100_at	CF404013	TC51810	Q8S569	Phosphoenolpyruvate carboxylase	Carbohydrate metabolism	2	3.07
1609470_at	CF203556	-	Q8LFZ9	Sucrase	Carbohydrate metabolism	5	3.04
1614023_at	CF414667	-	P46275	Fructose-1,6-bisphosphatase	Carbohydrate metabolism	2	3.01
1618726_at	CF211103	TC60540	Q5JNJ1	Trehalose-6-phosphate synthase/phosphatase	Carbohydrate metabolism	4	3
1614982_at	CF211066	TC61602	Q9C9P3	GDP-mannose pyrophosphorylase	Carbohydrate metabolism	3	2.99
1616783_at	CF405837	TC58450	P93344	Aldehyde dehydrogenase	Carbohydrate metabolism	11	2.95
1616630_at	CF603093	TC56347	Q94LX9	Phosphoenolpyruvate carboxylase	Carbohydrate metabolism	16	2.95
1620905_at	CF215819	TC68052	Q6RK07	UDP-glucose dehydrogenase	Carbohydrate metabolism	21	2.89
1621861_at	CF209183	TC65564	Q94AS2	b-amylase	Carbohydrate metabolism	11	2.87
1613188_at	CA817889	TC70258	Q5BLY1	a-amylase	Carbohydrate metabolism	11	2.85
1608207_at	CB343787	TC63660	Q84V96	Aldehyde dehydrogenase	Carbohydrate metabolism	3	2.7
1611808_at	CF205006	TC67979	Q94EU9	b-amylase	Carbohydrate metabolism	9	2.69
1610410_at	CB342966	TC61245	O64733	Glucosyltransferase	Carbohydrate metabolism	9	2.67
1611851_at	BQ799617	TC52022	Q9FIK0	Phosphofructo-1-kinase	Carbohydrate metabolism	10	2.67
1609545_at	CF514819	TC52560	Q4R0T9	ADP-sugar diphosphatase	Carbohydrate metabolism	11	2.64
1617368_at	CF512540	-	E1313	Glucan endo-1,3-b-glucosidase	Carbohydrate metabolism	3	2.63
1615623_at	CF511813	TC55899	O64733	Glucose acyltransferase	Carbohydrate metabolism	2	2.63
1620375_at	CA814054	TC62155	Q8LK43	Glycogene synthase kinase-like kinase	Carbohydrate metabolism	7	2.58
1613601_at	CB978458	TC67353	O64927	Starch synthase	Carbohydrate metabolism	3	2.56
1618125_at	BQ798742	-	Q94KE3	Pyruvate kinase	Carbohydrate metabolism	16	2.52
1617941_at	CB914224	TC62494	O81505	Water dikinase	Carbohydrate metabolism	11	2.52
1621073_at	CB914439	TC55380	Q7XEL0	GDP-mannose-3",5"-epimerase	Carbohydrate metabolism	3	2.51
1620165_at	CA817563	TC56014	Q84YG5	Isoamylase	Carbohydrate metabolism	11	2.51
1613060_at	CF214238	TC53819	Q9M3B6	Pyruvate kinase	Carbohydrate metabolism	18	2.51
1620904_at	CF609568	TC58209	Q9SAD5	b-1,4-N-acetylglucosaminyltransferase	Carbohydrate metabolism	16	2.49
1611027_at	CB978747	TC56057	Q3L7K5	Invertase	Carbohydrate metabolism	20	2.49
1608995_at	BQ796616	TC54941	Q84NI6	a-galactosidase	Carbohydrate metabolism	11	2.48
1622806_at	CB009073	TC63769	Q6VWJ5	Fructokinase	Carbohydrate metabolism	1	2.48
1609510_at	CF513342	TC69905	Q0WV85	O-linked GlcNAc transferase	Carbohydrate metabolism	16	2.47
1609232_at	CA811215	TC56883	Q9ZVJ5	Phosphoglucomutase	Carbohydrate metabolism	15	2.45
1613514_s_at	CF202452	TC54941	Q9M442	a-galactosidase II	Carbohydrate metabolism	11	2.44
1613025_at	CF403382	TC69507	Q9SNY3	GDP-mannose 4,6 dehydratase 1	Carbohydrate metabolism	21	2.43
1614514_at	CF405361	TC66847	Q84V39	Glucan endo-1,3-b-glucosidase	Carbohydrate metabolism	2	2.42
1608156_at	CF207998	TC58210	Q9XEY7	Trehalase	Carbohydrate metabolism	11	2.4
1612056_at	BQ795970	-	Q5BMC5	Phosphomannose isomerase	Carbohydrate metabolism	19	2.39
1612295_at	CF512417	TC67968	Q5VMJ5	Pyrophosphate-dependent phosphofructo-1-kinase	Carbohydrate metabolism	15	2.38
1609079_at	BQ796278	TC60979	Q94KE3	Pyruvate kinase	Carbohydrate metabolism	13	2.36
1615874_at	CF403960	TC54126	Q93XR7	Fructose-6-phosphate,2-kinase\/fructose-2,6-bisphosphatase	Carbohydrate metabolism	2	2.35
1608883_at	CA818676	TC60515	Q94AA4	Pyrophosphate-dependent phosphofructo-1-kinase	Carbohydrate metabolism	15	2.34
1616002_s_at	CB345569	TC52261	Q8LL68	Aldolase	Carbohydrate metabolism	3	2.29
1620865_at	CB917214	TC66899	Q7XBE4	Enolase	Carbohydrate metabolism	11	2.29
1607147_at	CF404016	-	Q5BLY0	a-amylase	Carbohydrate metabolism	10	2.28
1607727_at	CB976321	TC57680	Q5IH14	Sucrose-6-phosphate phosphatase	Carbohydrate metabolism	11	2.26
1614707_at	BQ799313	TC53692	P32811	a-glucan phosphorylase	Carbohydrate metabolism	21	2.25
1610277_at	CF208016	TC70514	Q50HW6	b-1,3-glucuronosyltransferase	Carbohydrate metabolism	2	2.25
1621432_s_at	CD005042	TC52007	Q8VXZ7	a-galactosidase	Carbohydrate metabolism	10	2.18
1621053_at	CF414284	TC63955	Q6VWJ5	Fructokinase	Carbohydrate metabolism	3	2.16
1621719_at	CF404994	TC65554	Q8LGH6	Dihydrolipoamide S-acetyltransferase	Carbohydrate metabolism	3	2.14
1619373_at	CB920390	TC69024	P80572	Alcohol dehydrogenase	Carbohydrate metabolism	3	2.13
1614612_at	CF513589	TC63370	Q9LSG3	Glucose acyltransferase	Carbohydrate metabolism	2	2.13
1615252_at	BQ792622	TC60550	Q5N8H1	Hydrolase-like protein	Carbohydrate metabolism	3	2.08
1612568_at	CF405938	TC67425	Q9LIB2	Glycogen phosphorylase B	Carbohydrate metabolism	13	2.07
1614153_at	CF207979	TC54491	Q7EYK9	Glucose-6-phosphate 1-dehydrogenase	Carbohydrate metabolism	4	2.03
1621378_at	BQ794342	TC61809	Q42581	Ribose-phosphate pyrophosphokinase 1	Nucleotide metabolism	11	4.5
1607578_at	CF415519	TC56533	O22141	Nucleotide sugar epimerase	Nucleotide metabolism	2	4.09
1608708_at	CF211873	TC53982	Q9SU83	Nucleotide pyrophosphatase	Nucleotide metabolism	18	3.73
1616669_at	CF209174	TC54382	Q3EAE2	dTDP-4-dehydrorhamnose reductase	Nucleotide metabolism	3	3.45
1609246_s_at	CF206363	TC54199	Q655Y8	UDP-glucose 4-epimerase	Nucleotide metabolism	4	3.14
1607889_a_at	CB976234	TC58106	Q6IVK4	UDP-glucuronate decarboxylase 2	Nucleotide metabolism	4	2.55
1622819_at	BQ798887	TC59368	O22141	Nucleotide sugar epimerase	Nucleotide metabolism	2	2.52
1616344_at	CF209136	TC68545	Q6XP48	UDP-glucose 4-epimerase	Nucleotide metabolism	21	2.52
1614498_at	CF213286	TC57825	O65781	UDP-galactose 4-epimerase	Nucleotide metabolism	21	2.32
1620930_s_at	CF212327	TC51843	Q6IVK4	UDP-glucuronate decarboxylase 2	Nucleotide metabolism	4	2.21
1614184_at	CF604220	TC66293	Q9SA77	UDP-galactose 4-epimerase	Nucleotide metabolism	21	2.13
1618478_at	CF515277	-	O64749	UDP-galactose-4-epimerase	Nucleotide metabolism	20	2.11
1616383_at	CF609704	TC59968	Q8L9F5	dTDP-glucose 4-6-dehydratase	Nucleotide metabolism	21	2.06
1615814_at	CB920915	TC56030	Q7FAH2	Glyceraldehyde-3-phosphate dehydrogenase	Phosphate Metabolism	10	2.87
1622715_s_at	CA809281	TC51781	P12858	Glyceraldehyde-3-phosphate dehydrogenase	Phosphate Metabolism	3	2.45
1618277_at	CF568829	TC56963	Q8VWN9	Glyceraldehyde-3-phosphate dehydrogenase	Phosphate Metabolism	21	2.22
1616083_at	CB009608	TC51694	Q9ZR63	Hexose transporter (VvHT1)	Transport	2	12.37
1610527_at	CA815926	TC52979	Q84QH3	Sorbitol transporter	Transport	2	5.49
1615257_at	CB972713	TC65400	Q4U339	Hexose transporter (VvHT6)	Transport	15	4.7
1619691_at	CF211807	TC62520	Q4U339	Hexose transporter (VvHT6)	Transport	14	3.69
1613408_at	CB347178	TC66667	P93075	Sucrose transporter (BvST1)	Transport	11	2.92
1608991_at	CA816013	TC60060	Q8GTR0	Sugar transporter	Transport	10	2.86
1610298_at	CB972367	TC53493	Q8LES0	Golgi nucleotide sugar transporter (GONST) 4	Transport	2	2.71
1615697_at	AF021810	TC51724	Q4JLW1	Sucrose transporter (VvSuc27)	Transport	3	2.44
1611331_at	CF201541	TC69532	Q69M22	Golgi nucleotide sugar transporter (GONST) 4	Transport	7	2.2
1612481_at	CF213270	-	Q6ID34	Glycerol 3-phosphate transporter	Transport	4	2.03

### Hexose and triose phosphate transport

The transcript abundances of numerous hexose and triosephosphate transporters varied considerably over the course of berry development (Figure 8C) indicating that each may fulfill different transport roles. The transcript abundance for a VvHT1 (1616083_at, TC51694), a previously described hexose transporter (VvHT1) located at the sieve cell-companion cell interface in the phloem and thought to play a major role in providing energy (mainly from glucose) for cell division and cell growth during the early stages of berry development [99], was high during Phase I, but then declined rapidly during ripening; this is largely consistent with an earlier report [18]. A second hexose transporter, VvHT6 (1615257_at; TC65400) exhibited a peak in transcript abundance near the start of véraison (E-L stage 34), which correlated well with hexose accumulation in the berries (Figure 8A), indicating that this transporter may play a significant role in hexose accumulation during berry ripening. Another previously described sucrose transporter (VvSUC27; 1615697_at, TC51724) [100], exhibited decreased transcript abundance throughout berry development consistent with earlier observations.

A putative plastidic glucose transporter (1608991_at, TC60060) showed increased transcript abundance up to E-L stage 34 and then remained constant throughout berry ripening (Figure 8C). The transcript abundance of a putative plasma membrane sugar/polyol transporter (1613408_at, TC66667), which resembles the AtPLT5 gene from *A. thaliana *[101] and is also capable of hexose transport, increased gradually over the course of berry development. In addition, two transcripts encoding a plastidial phosphate translocator-like (PTL) protein (1619379_at, TC58801) and a plastidial triosephosphate/phosphate translocator, TPT (1622157_at, TC61733) [102] displayed similar expression patterns that peaked at E-L stage 34 and then declined with berry ripening. The observed patterns of expression of the plastidial glucose and triosephosphate transporters indicate that both glucose and triosephosphates may be mobilized as export products as a result of active starch metabolism in plastids of developing and ripening berries.

**Figure 9:**
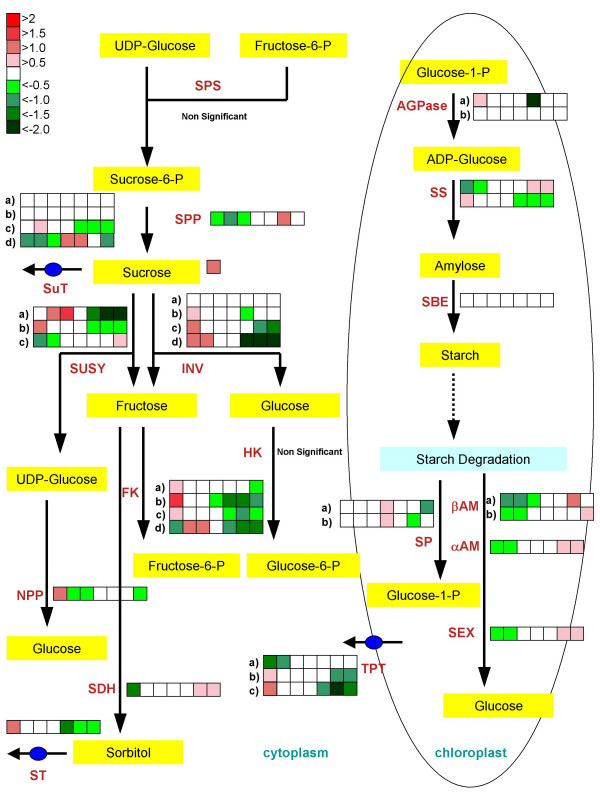
**Transcriptomic mapping of transcripts related sucrose and starch metabolism along berry development**. **SPS**: **s**ucrose **p**hosphate **s**ynthase-(1614674_at, TC60623), **SPP**: **s**ucrose **p**hosphate **p**hosphorylase – (1608257_at, TC68135) **SUSY**: **su**crose **sy**nthase – a) (1616700_at, TC53526) b) (1619223_s_at, TC52910) c) (1609402_at, TC62599) **INV**: **inv**ertase – a) (1620628_at, TC67908) b) (1611027_at, TC56057) c) (1612836_at, TC57719) d) (1611613_at, TC60693) **HK**: **h**exo**k**inase-(1611419_at – TC53318) **FK**: **f**ructo**k**inase – a) (1628006_at, TC63769), b) (1622282_at, TC54393), c) (1621053_at, TC63955), d) (1616255_at, TC57339) **SuT**: **su**crose **t**ransporter – a) (1620256_at, AF021808) b) (1622221_at, AF021809) c) (1615697_at, TC51724) d) (1615257_at, TC65400) **NPP**: **n**ucleotide **p**yro**p**hosphatase – (1620770_at, TC53085) **SDH**: **s**orbitol **d**e**h**ydrogenase – (1608527_at, TC58983) **ST**: **s**orbitol **t**ransporter – (1610527_at, TC52979) **AGPase**: **A**DP-**g**lucose **p**hosphatase – a) (1608393_at, TC64860) b) (1610928_at, TC64860) **SBE**: **s**tarch **b**ranching **e**nzyme – (1621790_at, TC65671) **SS**: **s**tarch **s**ynthase – a) (1615571_at, TC53551) b) (1613601_at, TC67353) **SP**: **s**tarch **p**hosphorylase – a) (1622120_at, TC54533) b) (1614707_at, TC53692) α **AM**: α-**am**ylase – (1613188_at, TC70258) β **AM**: β-**am**ylase – a) (1617124_at, TC67979) b) (1611808_at, CF205006) **SEX**: water dikinase – (1617941_at, TC62494) **TPT**: **t**riose **p**hosphate **t**ransporter – a) (1608991_at, TC60060) b) (1619379_at, TC58801) c) (1622157_at, TC61733). Each square from left to right corresponds to the expression of the probe sets from stage 31 through stage 38. Nonsignificant: Does not pass the ANOVA filter.

Finally, a sorbitol transporter (Figure 9) that has high homology with a cherry sorbitol transporter (PcSOT2) [103], has high transcript abundance early in fruit development as it does in cherry fruit. This transporter has high specificity for sorbitol as compared to its isomer, mannitol [103]. We were able to detect a sugar alcohol in our polar extracts using GC-MS, but were unable to distinguish whether it was sorbitol or mannitol. Further work will be done to distinguish sorbitol from mannitol. Note, however, that sorbitol has been detected in the sap of grapevines [104].

### Starch metabolism

Starch metabolism in developing and ripening grape berries is poorly understood. Starch synthase I catalyzes the elongation of glucans by the addition of glucose residues from ADP-glucose through the formation of α-1,4 linkages and is a major determinant for the synthesis of transient starch reserves in plants [105]. Our data indicate that starch metabolism is significant in berries. Starch concentrations declined significantly during Phase III of berry development; E-L stage 35, 36 and 38 were equal to 774 ± 57, 715 ± 54 and 554 ± 28 μg of glucose per g fresh weight of berry, respectively (mean ± SE).

Furthermore, the transcript abundance of numerous transcripts involved in starch metabolism changed during berry development. One plastidial soluble starch synthase Unigene (1615571_at, TC53551) displayed increasing transcript abundance, while a second Unigene (1613601_at, TC67353) displayed decreasing transcript abundance during berry development (Figure 8D). A transcript for the plastidial α-glucan, water dikinase (Gwd) gene (1617941_at, TC62494), which encodes an enzyme that is a regulator of starch mobilization and is essential for starch degradation [106], showed increased accumulation during berry development much like starch synthase I (1615571_at, TC53551). A second Gwd isogene (1617068_at, TC54621), showed peak transcript expression at E-L stage 35, but declined in fully ripe berries. Expression of plastidial α-1,4 glucan phosphorylase (Starch phosphorylase L isozyme, 1622120_at, TC54533), a starch mobilization enzyme that phosphorylates amylopectin to catalyze the release of glucose-1-phosphate, was nearly coordinate with the expression of this latter Gwd isogene. Finally, transcripts encoding the starch degrading enzymes, α-amylase (1613188_at, TC70258) and β-amylase (1617124_at, TC67979), both showed increased abundance during berry development (Figure 8D). Grape berries are likely to contain intact and functional plastids at véraison and at later stages of ripeness as shown by *in situ *fixation of exocarp and mesocarp cells [107].

Figure 9 summarizes the major pathways of hexose sugars and polysaccharide flux and putative transport processes in the developing berry as defined by the combined transcriptomic and metabolite analyses performed in this study. Abridged gene expression patterns for key regulatory genes involved in both sucrose and starch metabolism are shown. One can easily visualize the coordinate transcript expression patterns for the entire pathway along berry development. It is not apparent from this analysis why fructose concentrations would be higher than glucose in berries. This indicates that the regulation of these hexoses by hexokinase genes, whose transcripts did not significantly change (data not shown), is more complex than what can be discerned from a simple examination of transcript profiles.

### Photosynthesis and carbon assimilation

During berry development transcripts encoding proteins associated with photosynthesis-related functions are strongly expressed during the flowering stage and the so-called "herbaceous phase" or Phase I of berry development with expression declining during the later stages of berry maturation [17,18]. In our data, around 100 Unigenes with photosynthesis-related functions were identified with most displaying a steady or transient decline in the transcript abundance across berry development (Additional file 5, Table 7). Similarly, transcripts encoding enzymes with roles in carbon assimilation also exhibited a declining pattern of expression. For instance, Unigenes encoding Calvin cycle enzymes such as glyceraldehyde-3-phosphate dehydrogenase (1615814_at, TC56030; 1622715_s-at, TC51781), phosphoribulokinase (1614716_at, TC58640), transketolase (1618061_a_at, TC52548) as well as ribulose biphosphate carboxylase/oxygenase small subunit (1612848_x_at, TC64044) were highly expressed and then declined during Phase III of berry development consistent with previous reports [18].

**Table 7:** Transcripts (TFR pool) related to Energy metabolism within specific sub-sections

Probeset ID	GenBank Annotation	VvGI5	Uniprot ID	Gene Name Description	Function	Profile	Fold Change
1612882_at	CD720949	TC64621	A5BS41	ATP-dependent transmembrane transporter	ATP binding	2	5.55
1612645_at	CB344170	TC55708	P32980	ATP synthase	ATP binding	3	3.59
1616533_at	CB339497	TC62259	P31853	ATP synthase B' chain	ATP binding	12	2.88
1618182_at	CF604629	TC63054	P19023	ATP synthase beta chain	ATP binding	21	2.23
1607759_at	CD798264	TC67523	Q43433	Vacuolar ATP synthase subunit B isoform 2	ATP binding	11	2.16
1620500_at	CB349662	CB349662	Q67IU5	Ribulose 1,5-bisphosphate carboxylase small subunit	Carbon dioxide fixation	3	19.73
1613936_x_at	CF568996	CF568996	O22077	Ribulose bisphosphate carboxylase small chain	Carbon dioxide fixation	3	2.36
1616918_s_at	CB345541	TC56391	Q40281	Rubisco activase	Carbon dioxide fixation	3	2.22
1619681_at	CD799678	TC70996	Q9C5C7	Rubisco expression protein	Carbon dioxide fixation	11	2.18
1612848_x_at	CF202280	CF202280	P10795	Ribulose bisphosphate carboxylase	Carbon dioxide fixation	3	2.13
1620551_s_at	CB339855	TC56836	O98997	Rubisco activase	Carbon dioxide fixation	3	2.13
1610491_at	CD010750	TC57419	Q8LF17	Ribulose-1,5-bisphosphate carboxylase/oxygenase	Carbon dioxide fixation	12	2.13
1616847_s_at	CA816751	TC68454	O22077	Ribulose bisphosphate carboxylase small chain	Carbon dioxide fixation	3	2.12
1616435_at	CB974220	TC68219	P08927	RuBisCO subunit binding-protein beta subunit	Carbon dioxide fixation	3	2.1
1622299_s_at	CK136935	CK136935	Q9LKH8	NADPH-protochlorophyllide oxidoreductase	Chlorophyll biosynthesis	3	5.3
1619717_at	CF210684	TC59048	Q9SDT1	NADPH:protochlorophyllide oxidoreductase	Chlorophyll biosynthesis	3	4.79
1606624_at	CF606923	TC64589	Q43082	Porphobilinogen deaminase	Chlorophyll biosynthetic process	1	2.5
1617935_at	CB974545	TC68056	Q7YJS8	NADH dehydrogenase 49kDa subunit	Complex 1	16	5.55
1611418_at	CB342953	TC56161	Q7YJ08	NAD(P)H-quinone oxidoreductase	Complex 1	14	5.09
1609373_at	CD800734	TC60468	Q6KGY1	NADH dehydrogenase	Complex 1	14	3.05
1614095_at	CF606244	TC62268	P06261	NAD(P)H-quinone oxidoreductase	Complex 1	21	2.83
1609421_at	BQ795266	TC62811	O65414	NADH dehydrogenase	Complex 1	11	2.75
1617757_at	CA818465	TC58524	Q6YSN0	NADH dehydrogenase	Complex 1	21	2.42
1612005_s_at	CB004075	TC64671	Q68S01	NADH dehydrogenase	Complex 1	3	2.27
1610869_at	CF515388	TC56269	Q8H2T7	NADH dehydrogenase subunit	Complex 1	16	2.23
1610347_s_at	CF202826	CF202826	Q0ZIW2	NAD(P)H-quinone oxidoreductase	Complex 1	10	2.1
1609391_s_at	CF404650	TC53103	Q41001	Copper Binding Protein	Copper ion binding	2	26.11
1620588_at	CD801157	CD801157	Q8LED5	Mavicyanin	Copper ion binding	6	25.27
1621220_at	CB919187	TC59624	Q9M510	Dicyanin	Copper ion binding	11	17.36
1610220_at	CB973621	TC68272	O81500	Copper Binding Protein	Copper ion binding	3	14.36
1617350_at	CB975555	TC58747	Q39131	Copper Binding Protein	Copper ion binding	3	10.21
1611332_at	CF371813	TC59560	Q653S5	Blue copper binding protein (bcb)	Copper ion binding	1	7.64
1607270_at	CB923224	TC60083	Q9ZRV5	Copper Binding Protein	Copper ion binding	2	5.87
1620744_at	CF403966	TC65998	P17340	Plastocyanin, chloroplast precursor	Copper ion binding	3	4.41
1617046_at	CF512505	TC54856	O23230	Trichohyalin	Copper ion binding	3	2.23
1609233_at	CF512410	TC54170	Q84RM1	Copper Binding Protein	Copper ion binding	2	2.21
1618207_at	CB347324	TC52865	Q9C540	Cytochrome 561	Electron carrier activity	3	7.45
1612624_at	CB974055	TC58854	P06449	Apocytochrome f	Electron carrier activity	21	5.32
1611598_at	CB970208	TC60594	Q9ZSR3	Cytochrome b-561	Electron carrier activity	2	4.54
1606617_at	CF608010	TC65350	O23344	Electron transport	electron carrier activity	3	2.49
1606704_s_at	CF200937	CF200937	P59702	Cytochrome b559 alpha subunit	Electron carrier activity	21	2.33
1615927_s_at	CB972155	TC55109	Q6Q8B8	Chloroplast ferredoxin I	Electron carrier activity	3	2.23
1607800_at	CB972521	TC52149	Q84WN3	Cytochrome c oxidoreductase	Electron transport	2	17.79
1620504_at	CB342755	TC52829	Q84WN3	cytochrome c oxidoreductase	Electron transport	7	14.78
1618535_at	CA818656	CA818656	Q6V5G1	Cu2+ plastocyanin	Electron transport	13	6.25
1615046_at	CF210436	TC59116	P41346	Ferredoxin--NADP reductase	Electron transport	3	5.61
1614266_at	BQ792322	TC57184	Q49KU9	Cytochrome c heme attachment protein	Electron transport	16	4.17
1613158_at	CB349843	CB349843	O47437	Cytochrome c oxidase	Electron transport	16	3.56
1612766_s_at	CF569219	CF569219	Q5PY86	NADH-cytochrome b5 reductase	Electron Transport	3	3.32
1619756_at	CB003378	TC59085	Q9LYC6	Glutaredoxin	Electron transport	14	2.88
1620991_at	CB344999	TC58191	O24068	Cytochrome oxidase subunit 3	Electron transport	16	2.26
1606445_a_at	CF512668	TC62694	P26291	Cytochrome B6-F complex iron-sulfur subuni	Electron transport	3	2.16
1608372_at	CF208491	TC51964	Q6K7S7	Cytochrome c biogenesis	Electron transport	9	2.1
1621402_a_at	CF213496	TC53161	P00051	Cytochrome c	Electron transport	11	2.01
1607356_at	CB911288	TC67262	Q8LCF6	Hypothetical Protein	ENERGY	11	12.28
1611972_s_at	CF519112	TC53292	A4X6H5	Cytochrome b	ENERGY	3	11.63
1615762_at	CD798079	TC66865	O80763	Hypothetical Protein	ENERGY	10	4.82
1616241_at	CD797326	CD797326	Q8VYC5	Hypothetical Protein	ENERGY	16	4.08
1609285_at	CF414528	TC57440	Q9FFT2	Hypothetical Protein	ENERGY	2	3.44
1611820_at	CB914713	TC69253	O80763	Hypothetical Protein	ENERGY	3	3.09
1606562_at	CF404246	TC59415	A3J369	Nitrilase 1	ENERGY	11	3.04
1621817_at	CB978007	TC64650	O80763	Hypothetical Protein	ENERGY	3	3
1614875_at	CF518552	TC69033	A5AU55	Hypothetical Protein	ENERGY	11	2.73
1622517_at	CB970523	TC54809	Q8W4Z5	Hypothetical Protein	ENERGY	3	2.39
1621903_at	CF404558	TC62294	Q9FE29	Hypothetical Protein	ENERGY	13	2.23
1612648_at	CD798203	CD798203	O80763	Hypothetical Protein	ENERGY	21	2.15
1622345_at	CB970837	TC55633	Q7XTZ0	Mandelonitrile lyase	Flavoprotein	2	4.87
1606948_at	CF404230	CF404230	Q01JW7	Mandelonitrile lyase	Flavoprotein	2	3.68
1622745_at	BQ796736	TC58626	Q8L5Q7	Quinone oxidoreductase	FMN binding	15	19.63
1615481_at	CB973026	TC62178	Q9ZSP7	Cytochrome b5 DIF-F	Iron ion binding	3	2.57
1606727_at	BQ799998	TC62672	Q58IV4	Phytochrome C	Light Signaling	10	2.38
1617604_at	CF609932	TC59809	Q94BM7	Phytochrome A supressor spa1 protein	Light Signaling	11	2.25
1611135_at	CB983077	TC51911	Q9SG92	Alpha-hydroxynitrile lyase	Lyase activity	11	2.92
1622108_at	CF405863	TC56579	Q9SU40	Monocopper oxidase	Multicopper oxidase family	3	23.07
1621115_at	CF609165	TC64136	Q9SU40	Monocopper oxidase	Multicopper oxidase family	1	20.1
1617992_a_at	CF213671	TC60094	P51132	Ubiquinol--cytochrome-c reductase-like protein	Oxidative Phosphorespiration	11	2.18
1611597_at	CB918250	CB918250	Q8LDU4	Red chlorophyll catabolite reductase	Oxidoreductase activity	12	2.03
1613786_at	CD714955	TC57282	Q6QY10	P700 chlorophyll a apoprotein A1	Photosystem I	21	8.65
1611364_at	CF211293	TC52528	Q9XQB4	Reaction center subunit III	Photosystem I	3	7.67
1611464_at	CF215949	TC59235	Q9XF85	Lhca5 protein	Photosystem I	3	7.21
1621532_at	CB973721	TC64270	Q84QE6	Reaction center subunit X psaK	Photosystem I	3	7.15
1619903_at	CD720479	TC65556	Q40512	Light-harvesting chlorophyll a/b-binding protein	Photosystem I	3	6.96
1619629_at	CB340944	TC66352	Q5DNZ6	Chlorophyll a-b binding protein	Photosystem I	3	6.59
1622534_at	BQ799942	TC53444	Q84U30	Photosystem I-N subunit	Photosystem I	3	6.45
1611733_s_at	BQ797982	TC52546	Q70PN9	Reaction centre PSI-D subunit precursor	Photosystem I	3	6.44
1616560_at	CA817733	TC62550	Q84WT1	Light-harvesting chlorophyll a/b binding protein	Photosystem I	3	5.9
1611515_s_at	CB343423	TC57304	O65101	Reaction center subunit VI	Photosystem I	3	5.33
1618370_at	CF510718	TC57721	Q9SUI4	Reaction center subunit XI	Photosystem I	3	5.31
1617771_at	CF414158	TC58342	Q8RVJ8	Reaction centre subunit IV	Photosystem I	3	4.87
1618127_at	CB968637	TC62932	Q9SY97	Chlorophyll a/b-binding protein	Photosystem I	3	4.74
1614409_at	CA817387	TC55189	P13869	Chlorophyll a-b binding protein	Photosystem I	3	4.65
1614593_at	CF511805	TC52379	Q00321	CP29 polypeptide	Photosystem I	3	4.43
1611924_at	CA817406	TC63702	Q646H3	Reaction center V	Photosystem I	3	4.06
1611161_at	CF210442	TC54044	Q9ZU86	Expressed protein	Photosystem I	3	3.09
1622302_s_at	CF207602	TC54765	Q40459	Oxygen-evolving enhancer protein 1	Photosystem I	3	2.8
1610245_at	CF209798	TC53968	Q41424	Chlorophyll a/b binding protein	Photosystem II	3	15.82
1615822_at	CF208321	TC52049	Q9XQB1	LHCII type III chlorophyll a/b binding protein	Photosystem II	3	10.43
1608311_at	CF202519	CF202519	Q7M1K9	Chlorophyll a/b-binding protein	Photosystem II	3	10.24
1616940_s_at	CB348709	TC52113	Q7M1K9	Chlorophyll a/b-binding protein	Photosystem II	3	9.95
1618116_s_at	BQ798823	TC55659	Q32291	Chlorophyll A/B binding protein precursor	Photosystem II	3	7.28
1611860_at	CF209952	TC57521	Q9XQB6	Chlorophyll a/b-binding protein CP24	Photosystem II	3	6.6
1612085_at	CF413799	TC54542	Q41387	Reaction center W protein	Photosystem II	3	5.79
1621038_at	CF372077	TC57214	O64448	Light harvesting chlorophyll a/b-binding protein precursor	Photosystem II	3	5.77
1618679_s_at	CB343106	TC52042	Q9BBT1	44 kDa reaction center protein	Photosystem II	16	5.41
1610203_at	CD009386	TC56267	Q7YJY8	Photosystem Q(B) protein	Photosystem II	21	5.11
1613991_at	CF510955	TC53743	P80470	Core complex proteins psbY	Photosystem II	3	4.77
1607516_at	CB972913	TC53930	Q9LRC4	Oxygen evolving enhancer protein 1 precursor	Photosystem II	3	4.66
1613428_at	CF207158	TC52084	Q5PYQ5	Chloroplast oxygen-evolving enhancer protein	Photosystem II	3	4.47
1607961_at	CF415716	TC57429	P31336	5 kDa protein	Photosystem II	3	4.16
1614598_at	CF373065	TC61762	Q9XQB2	Chlorophyll a/b binding protein CP29	Photosystem II	3	4.06
1613691_s_at	CF511746	TC54828	P27518	Chlorophyll a-b binding protein 151	Photosystem II	3	3.84
1613773_s_at	BQ799145	TC63656	Q41387	Reaction center W protein	Photosystem II	3	3.34
1618031_s_at	CF404451	TC53833	Q8GV53	10 kDa protein	Photosystem II	3	3.28
1621351_s_at	CB340283	TC53732	Q40961	Light-harvesting chlorophyll a/b-binding protein precursor	Photosystem II	3	3.2
1613494_s_at	CA813944	TC55522	Q9SLQ8	Oxygen-evolving enhancer protein 2	Photosystem II	3	3.05
1617605_at	CF513977	TC55526	Q8HS34	CP47 protein	Photosystem II	18	2.99
1618274_at	CB972471	TC55538	Q4FFQ9	Phosphoprotein	Photosystem II	21	2.92
1610144_at	CB342508	TC53591	Q9MTN0	Uncharacterized 6.9 kDa protein in psbD-trnT intergenic region	Photosystem II	15	2.88
1611582_s_at	CB970190	TC70959	Q02060	22 kDa protein	Photosystem II	3	2.69
1607926_at	CF202256	CF202256	Q9AR57	Putative membrane protein	Photosystem II	2	2.56
1621978_at	CB837910	TC56626	Q9M3M7	Uncharacterized protein	Photosystem II	16	2.31
1607803_at	CB975690	TC52112	Q06364	26S proteasome non-ATPase regulatory subunit 3	Photosystem II	21	2.31
1619523_at	CB969438	TC67627	Q952R1	Succinate dehydrogenase	Succinate dehydrogenase activity	15	2.24

### Circadian cycles

Circadian clocks are signaling networks that enhance an organism's growth, survival, and competitive advantage in rhythmic day/night environments [108]. The plant circadian clock modulates a wide range of physiological and biochemical events, such as stomatal and organ movements, photosynthesis and induction of flowering. A model of circadian rhythm based upon activities of several enzymes has been created involving transcription factors such as CIRCADIAN CLOCK-ASSOCIATED 1 (CCA1) or pseudo-response regulators such as PRR7 [108]. Transcripts for Unigene (1616834_at, TC54726) encoding CCA1 were repressed during the early stages of berry development, but increased in abundance at E-L stage 36. In contrast, one Unigene (1608006_at, TC51808) related to the two-component response regulator APRR7 had a transient peak of expression in the early stages of berry development. This result is consistent with the position and function of these proteins in the circadian clock. Indeed, APRR7 represses CCA1 activity in *Arabidopsis thaliana*. In grape, these correlations in the transcript abundance indicate the operation of the circadian clock machinery throughout berry development. In addition, those genes are thought to enhance starch mobilization, consistent with previous observations made during Phase III of berry development [109].

### Pathogen and disease resistance related proteins

Pathogen-related (PR) proteins are the most abundant class of proteins present in wine and can negatively affect the clarity and stability of wine [110]. During berry development, PR genes are expressed highly throughout various stages of berry growth. Around 30 Unigenes encoding different classes of PR genes were identified with a two-fold ratio or greater expression change (Additional file 5, Table 8). Interestingly, four Unigenes encoding PR1 protein were highly expressed during early berry development, but then declined for the remainder of berry development. PR1 protein is regarded as one of the main down-stream responses of the salicylic acid signaling that plays an important role in Systemic Acquired Resistance. Salycylic acid is thought to accumulate just before véraison, which correlates well with the PR1 mRNA and protein expression [111]. The two main PR proteins that have a significant role in the defense against invading fungal pathogens are β-1,3-glucanase (PR2) and chitinase (PR3) [112]. Five Unigenes encoding β-1,3-glucanase were transiently expressed at different periods of berry development. Unigenes encoding various chitinases were also identified that displayed similar mRNA expression patterns. Some chitinase genes exhibit strong homologies with a chitinase previously observed in grape berry [111]. Another PR protein, which may play a role in grape berry defense, is thaumatin protein (PR5) [113]. Eight Unigenes encoding PR5 proteins were identified and their respective expression patterns span all stages of berry development. Taken together, the expression patterns revealed that these defense-related gene products and enzymes are expressed across all stages of berry development. Such a Systemic Acquired Resistance strategy probably minimizes pathogen invasion as previously suggested [114].

**Table 8:** Transcripts (TFR pool) related to Pathogenesis-Related proteins within specific sub-sections

Probeset ID	GenBank Annotation	VvGI5	Uniprot ID	Gene Name Description	Category	Profile	Fold Change
1613471_at	CF215857	TC59306	Q9SW05	Pathogenesis-related protein	PR1	3	7.83
1611058_at	CA814153	TC67060	Q7XAJ6	Pathogenesis related protein 1	PR1	3	5.76
1613816_x_at	CF074673	TC56938	Q7XAJ6	Pathogenesis related protein 1	PR1	21	4.74
1618533_at	CB970020	TC55782	Q40374	Pathogenesis related protein 1	PR1	2	2.31
1615595_at	AF239617	AF239617	Q9M563	β-1,3-glucanase	PR2	11	10.99
1620496_at	CF214365	TC66187	Q8VY12	β-1,3-glucanase	PR2	15	2.3
1608203_at	CF511734	TC64974	Q94EN5	β-1,3-glucanase	PR2	2	2.17
1616183_at	CF405742	TC62849	Q94G86	β-1,3-glucanase	PR2	14	2.17
1610324_a_at	CB346041	TC67051	Q8L868	β-1,3-glucanase	PR2	12	2.05
1621319_s_at	CB981122	TC70080	Q7XAU6	Chitinase IV	PR3	10	299.34
1613461_s_at	AF532966	AF532966	Q7XAU6	Chitinase IV	PR3	10	162.58
1607557_at	CF202548	CF202548	Q7XAU6	Chitinase IV	PR3	10	149.38
1614551_at	CB343715	TC51734	Q6JX04	Chitinase	PR3	2	49.79
1616064_at	CF205270	CF205270	O24531	Chitinase IV	PR3	11	20.19
1621583_at	CF404733	TC62834	Q6JX04	Chitinase	PR3	1	4.93
1620111_at	CF568854	CF568854	Q6JX04	Chitinase	PR3	2	3.36
1606625_at	CF603972	TC64563	Q7XB39	Chitinase IV	PR3	19	3.21
1620518_at	CF201341	CF201341	O81228	Pathogenesis related protein 4	PR4	15	43.11
1618835_s_at	BQ797163	TC58333	O81228	Pathogenesis related protein 5	PR4	15	25.9
1612160_at	CF415249	TC64611	P50699	Thaumatin	PR5	3	34.49
1618871_at	CF510551	TC55284	Q82L96	Thaumatin	PR5	3	15.65
1616617_at	AF195654	AF195654	Q9SNY0	Thaumatin	PR5	11	11.86
1607225_at	CB914105	TC65548	O65638	Thaumatin	PR5	11	8.51
1614746_at	CF214284	TC53053	Q7XST4	Thaumatin	PR5	2	5.27
1607708_at	CF413841	TC63177	Q9LZL8	Thaumatin	PR5	2	4.22
1606517_at	CB347191	TC62530	Q8LBL4	Thaumatin-like protein	PR5	14	3.51
1622374_at	CB920589	TC56535	Q41350	Thaumatin	PR5	2	3.34
1613999_x_at	CF202364	CF202364	Q84S31	Chitinase III	PR8	2	4.57

### Quantitative real-time RT-PCR

**Figure 10:**
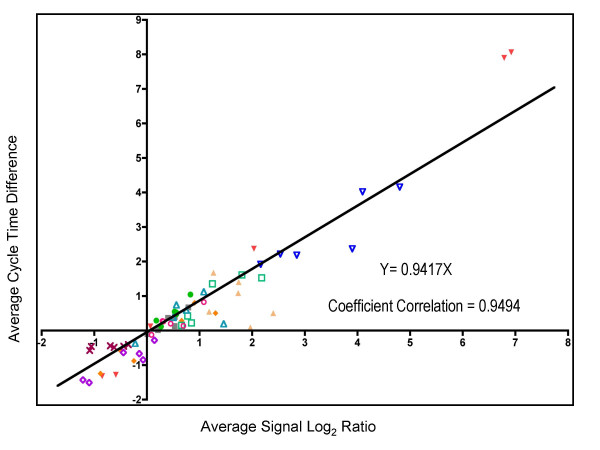
**Quantitative real-time RT-PCR of eleven transcripts**. Comparison between the gene expression ratios reported by the Affymetrix GeneChip^® ^genome array and by real-time RT-PCR. Data were from 11 probe sets across seven developmental stages. The difference in the number of PCR cycles required to produce the same amount of product is plotted against the log_2 _expression ratio averaged over the first time point. The linear regression line was constrained to pass through the origin. Grey solid square (1615402_at, TC56083)-ferulate-5-hydroxylase, Apricot solid triangle (1606794_at, TC63891)-osmotin precursor, red solid triangle (1616700_at, TC53526)-sucrose synthase, orange solid diamond (1607760_at, TC51695) flavonoid-3'5'-hydroxylase, light green solid round (1611650_at, TC57228)-WRKY7, dark green open square (1616880_at, TC54034)-cinnamoyl alcohol dehydrogenase, dark blue open triangle (1613896_at, TC62182)-nitrate/chloride transporter), blue open triangle (1615722_s_at, TC51776)-aquaporin PIP1.1, lavender open diamond (1611342_at, TC55943)-serine/threonine kinase, pink open circle (1612132_s_at, TC68311)-protein phosphatase 2C, brown cross (1614931_at, TC61058)-MYB transcription factor.

To validate expression profiles obtained using the Affymetrix GeneChip^® ^Vitis genome array, quantitative real-time RT-PCR was performed on 11 genes using gene-specific primers [Additional file 5, Table 3]. Transcript abundance patterns were calculated along the entire course of berry development. Linear regression ([microarray value] = a[RT-PCR value]+b) analysis showed an overall correlation coefficient of 0.94 indicating a good correlation between transcript abundance assessed by real-time RT-PCR and the expression profiles obtained with the GeneChip^® ^genome arrays (Figure 10).

## Conclusion

Our large-scale transcriptomic analysis demonstrated that nearly a third (28%) of genes expressed in berries exhibited at least two-fold or greater change in steady-state transcript abundance over the course of seven stages of grape berry development. Approximately two-thirds (64%) of these Unigenes could be assigned a functional annotation with the remaining one-third having obscure or unknown functions. Twenty distinct patterns of expression were resolved in order to illustrate the complex transcriptional regulatory hierarchies that exist to orchestrate the dynamic metabolic, transport, and control processes occurring in developing berries. We provided evidence that phytohormone biosynthesis and responses, particularly for ABA, ethylene, brassinosteroids, and auxins, as well as calcium homeostasis, transport, and signaling processes play critical roles in this developmental process. We also demonstrate that the expression and regulation of genes involved in cell wall biosynthesis and expansion, as well as genes involved in the biosynthesis, transport, and regulation of the phenylpropanoid and flavonoid pathways undergo dynamic changes throughout the course of berry development. Our analysis has revealed candidate genes that may participate in the production of different classes of aroma producing compounds. We have also demonstrated coordinate regulation of transcripts and the accumulation of key metabolites including tartrate, malate, and proline during berry development. A close examination of the behavior of gene expression patterns of genes involved in sugar and starch metabolism indicate that plastidial starch reserves are mobilized to fuel the production of hexose sugars during the ripening and maturation phase (Phase III) of berry development. Finally, our findings provide the first functional genomic information for hundreds of genes with obscure functions that can be exploited for hypothesis testing by traditional functional assays to improve our understanding of the complex developmental processes present in grape berries and to ultimately utilize this information to improve quality traits of wine grapes.

## Methods

### Plant Materials

Six twenty-year-old Cabernet Sauvignon (*Vitis vinifera *L.) vines grown on St. George rootstock were used during 2004 for this study. The vines were located at the Shenandoah Vineyard in Plymouth, CA, on a hillside row located in the middle of the vineyard. All plants were equipped with a drip irrigation system and watered daily to keep their water status high. Mid-day stem water potentials were measured weekly with a pressure chamber on two mature leaves per plant for a total of 6 vines [115]. For each measurement, a single leaf per plant was tightly zipped in a plastic bag to eliminate transpiration and covered with aluminum foil to deflect light and heat. After two hours of equilibration time, the petiole was cleanly cut and carefully threaded through a rubber gasket in the lid of a pressure chamber (3005 Plant Water Status Console, Soilmoisture Equipment Corp., Santa Barbara, CA, USA). The foil was removed before sealing the bagged leaf in the chamber. The balancing pressure required to visibly push stem xylem sap to the cut surface was recorded.

Two grape clusters were sampled weekly from each plant (*n *= 6), one from the south (sunny) and one from the north (shady) side of the plant. The clusters were pooled together for each plant in order to avoid light and temperature effects. Berry development was characterized by monitoring berry diameter, total soluble solids and titratable acidity. The berry diameter was measured with a micrometer for fifteen randomly selected berries per each of two clusters and an average berry size was computed per vine (*n *= 6). Total soluble solids (°Brix) were assayed (two technical replicates) with a refractometer (BRIX30, USA) from juice crushed from harvested berries from two clusters per vine (*n *= 6) to estimate total sugar content. Titratable acidity (g/L) of the grape juice was measured by titration to an endpoint of pH 8.4 with a strong base. The same number of repetitions as in °Brix measurements was used.

### RNA extraction and microarray hybridization

Total RNA was extracted from berries finely ground in liquid nitrogen using Qiagen RNeasy Plant MidiKit columns (Qiagen Inc., CA) as previously described [116]. The total RNA was further purified using a Qiagen RNeasy Plant Mini Kit (Qiagen, Valencia, CA) according to the manufacturers' instructions. RNA integrity was confirmed by electrophoresis on 1.5% agarose gels containing formaldehyde and quality was confirmed by analysis on an Agilent 2100 Bioanalyzer using RNA LabChip^® ^assays according to the manufacturer's instructions. mRNAs were converted to cDNAs using oligo dT primer containing a T7 RNA polymerase promoter sequence and reverse transcriptase. Biotinylated complementary RNAs (cRNAs) were synthesized *in vitro *using T7 RNA polymerase in the presence of biotin-labeled UTP/CTP, purified, fragmented and hybridized in the GeneChip^® ^*Vitis vinifera *Genome Array cartridge (Affymetrix^®^, Santa Clara, CA). The hybridized arrays were washed and stained with streptavidin phycoerythrin and biotinylated anti-streptavidin antibody using an Affymetrix Fluidics Station 400. Microarrays were scanned using a Hewlett-Packard GeneArray^® ^Scanner and image data was collected and processed on a GeneChip^® ^workstation using Affymetrix^® ^GCOS software.

### Microarray data processing

Three biological replicates per experiment were processed to evaluate intra-specific variability. Expression data were processed by RMA (Robust Multi-Array Average) [117] using the R package affy [118]. Specifically, the RMA model of probe-specific background correction was first applied to the PM (perfect match) probes. These corrected probe values were normalized via quantile normalization and a median polish method was applied to compute one expression measure from all probe values. Data quality was verified by digestion curves describing trends in RNA degradation between the 5' end and the 3' end in each probe set. Differentially expressed genes throughout berry development were determined by ANOVA on the RMA expression values [118]. A multiple testing correction [22] was applied to the *p*-values of the F-statistics to adjust the false discovery rate. Genes with adjusted *p*-values < 0.05 were extracted for further analysis. Genes having a two-fold ratio (TFR) or greater between at least two time points along berry development were selected for further analyses. The RMA expression data (experiment Vv5) have been deposited in PLEXdb [119].

### Microarray data analysis

Clustering of co-regulated genes was performed using the MultiExperimentViewer software part of the TM4 software package (MEV3.1) developed by TIGR [120]. TFR Unigenes were clustered via the Pavlidis Template Matching (PTM) algorithm [24]. The twenty template profiles were selected (by us) as representatives of biological processes occurring during berry development (Additional File 4). The Pearson correlation coefficient between each Unigene and each template profile was used to determine cluster membership: correlation measures greater than 0.75 corresponded to a good match. If genes were well correlated with more than one template profile, the gene was assigned to the cluster with which it had greatest correlation. The *p*-values associated to the hypothesis test of each correlation coefficient (null hypothesis is that the correlation is zero) were calculated and a multiple testing correction (Benjamini and Hochberg) was applied. Only genes with adjusted *p*-values ≤ 0.05 and correlations greater than 0.75 were placed into clusters

### Unigene Annotation and Functional Analysis

Unigene annotation was updated by nucleotide sequence query of the probe consensus sequence against the UniProt/TrEMBL, NCBI-nr and TAIR protein databases using BLASTX (e-value < 1e-05). Functional categories were assigned automatically by amino acid homology to *Arabidopsis thaliana *proteins categorized according to the Munich Information Center for Protein Sequences (MIPS) Funcat 2 classification scheme [25]. Bibliographic searches were performed to assign functions to Unigenes exhibiting no homology with *Arabidopsis thaliana *proteins. Some annotation presented here will be subject to error due to the relatively correlative nature of these associations. It is expected that the annotated data presented here will be used for future hypothesis-driven research that can establish stronger functional analyses and annotations.

Attribution of the 20 clusters to the key developmental phases (I, II or III) (See Figure 6) was decided according to two criteria. The first one was to fit these phases with the time points used in this study. Stage 31 (Modified E-L System) was the only one belonging to the herbaceous phase (Phase I). The lag phase (Phase II) corresponded to stages 32 to 34. The maturation phase (Phase III) included stages 35 to 38. The second criterion was based on the time point at which the maximum average gene expression value was observed across the genes within each cluster. For instance, cluster 1 was included in the Phase I group, because the maximum average expression level was observed at stage 31. The same assignments were made in the other phases (II and III) (See Additional File 5: Table 1). To test for significant differences in the representation of Unigenes within each functional category per developmental phase (Phases I, II and III; see Figure 5), a Pearson's chi-squared test was used [121]. Three comparisons (Phase I against II; I against III and II against III) were performed and results are listed in Additional File 5: Table 2. Differences in frequency for each category between two stages were considered significant for a *p*-value < 0.05.

### Real Time PCR experiments

RNA was extracted and its integrity verified by standard procedures. cDNA was synthesized using an iScript cDNA Synthesis Kit (Bio-Rad Laboratories, Hercules, CA) according to the manufacturer's instructions with a uniform 1 μg RNA per reaction volume reverse-transcribed. Primers for genes (Additional File 5: Table 3) assayed by real-time PCR were selected using Primer3 software [122]. Quantitative real-time PCR reactions were prepared using an iTaq SYBR Green Supermix with ROX (Bio-Rad) and performed using the ABI PRISM^® ^7000 Sequence Detection System (Applied Biosystems, Foster City, CA). Expression was determined for triplicate biological replicates by use of serial dilution cDNA standard curves per gene. In order to assess the performance of the array in a biological context, we examined the transcript abundance of some candidate genes from Cabernet Sauvignon exhibiting changing expression patterns across the 7 time points of berry development. Real-time RT-PCR was performed with the ABI PRISM® 7000 Sequence Detection System (Applied Biosystems, Forster City, CA) under annealing conditions of 50°C for 1 minute and analyzed with ABI PRISM® 7000 SDS software. Analysis of relative gene expression was performed using the 2−ΔΔCT
 MathType@MTEF@5@5@+=feaafiart1ev1aaatCvAUfKttLearuWrP9MDH5MBPbIqV92AaeXatLxBI9gBaebbnrfifHhDYfgasaacPC6xNi=xH8viVGI8Gi=hEeeu0xXdbba9frFj0xb9qqpG0dXdb9aspeI8k8fiI+fsY=rqGqVepae9pg0db9vqaiVgFr0xfr=xfr=xc9adbaqaaeGacaGaaiaabeqaaeqabiWaaaGcbaGaeGOmaiZaaWbaaSqabeaacqGHsislcqqHuoarcqqHuoarcqqGdbWqdaWgaaadbaGaemivaqfabeaaaaaaaa@3316@ method [123]. The data were analyzed using the equation ΔΔC_*T *_= (C_T,Target _- C_T,HG_) Time *X *- (C_T,Target _- C_T,HG_) Time *0 *where Time *X *is the value at any time point and Time *0 *represents the 1X expression of the target gene normalized to ankyrin. Data were calculated from the calibration curve and normalized using the expression curve of an ankyrin gene (1612584_s_at; TC53110), whose mRNA presented an extremely low coefficient of variation (0.056, M Value = 0.1297) through microarray analysis [124].

### Metabolite extraction and derivatization

Polar metabolites were extracted and derivatized with a water/chloroform protocol according to previously established procedures [125]. Freeze-dried berry tissue (6 mg) was placed in a standard screw-cap-threaded, glass vial. The tube was then returned to the -80°C freezer until use. Frozen tubes were wrapped in parafilm and freeze-dried overnight. All tissue samples were kept frozen throughout the lyophilization procedure. Upon lyophilization, tubes were capped and returned to the freezer until extraction. The vials were allowed to cool back to room temperature before being handled. The extraction vials were not washed with a methanol/hexane rinse, but all caps and septa were. The vial was incubated in HPLC grade chloroform for 1 hour at 50°C in an oven. A volume of Millipore water was added (m/V) containing 25 mg/L of ribitol as an internal standard and the sample was re-incubated for an additional hour at 50°C. Finally, vials were allowed to cool to room temperature and then spun down at 2,900 × g for 30 minutes. One mL of the polar phase was dried down in a vacuum concentrator. Polar samples were derivatized by adding 120 μL of 15 mg mL^-1 ^of methoxyamine HCl in pyridine, incubated at 50°C for 30 minutes and sonicated until all crystals disappeared. After that, 120 μL of MSTFA + 1% TMCS were added, incubated at 50°C for 30 minutes and immediately submitted for analysis with a Thermo Finnigan Polaris Q230 GC-MS (Thermo Electron Corporation, San Jose, CA, USA). The inlet and transfer lines were held at 240°C and 320°C, respectively. Separation was achieved with a temperature program of 80°C for 3 min, then ramped at 5°C min^-1 ^to 315°C and held for 17 min, using a 60 m DB-5MS column (J&W Scientific, 0.25 mm ID, 0.25 μm film thickness) and a constant flow of 1.0 ml min^-1^. Derivatized samples (120 μL) were transferred to a 200 μL silanized vial insert and run at an injection split of 200:1 to bring the large peaks to a concentration within the range of the detector. Identity of all organic acids, sugars and amino acids were verified by comparison with standards purchased from Sigma-Aldrich (St. Louis, MO, USA).

### Metabolite data processing

Metabolites were identified from the chromatograms using two different software packages: AMDIS (2.64, United States Department of Defense, USA) and Xcalibur (1.3; Thermo Electron Corporation). The software matched the mass spectrum in each peak against three different metabolite libraries: NIST ver. 2.0 library [126], T_MSRI_ID library of the Golm Metabolome Database [127] and our own custom-created UNR library (V1) made from more than 50 standards bought from Sigma-Aldrich. Quantification of the area of the chromatogram peaks was determined using Xcalibur and normalized as a ratio of the area of the peak of the ribitol internal standard.

### Starch determination

Starch assays were performed according to Dubois et al. [128]; 100 mg of berry powder from E-L stages (35 to 38) were finely ground and incubated in 5 mL of methanol (80/20; v/v) at 80°C for 40 min. This step eliminates soluble sugars. The methanol extract was removed and the pellet was washed twice with distilled water. The remaining pellet was incubated overnight in 1.2 mL of acetate buffer (40 mM sodium acetate, 60 mM acetic acid) and 0.2 mL of enzymes solution (3 units of amyloglucosidase and 0.25 units of α-amylase); 0.5 mL of the supernatant was mixed with 0.5 mL of water and 1 mL of phenol (5/95; v/v). Thereafter, 5 mL of concentrated sulfuric acid was added and the solution was left to cool for 15 min. Glucose was measured by its absorbance at 483 nm and expressed in terms of μg of glucose per g fresh weight of berry sample. Calibration of the concentration of glucose was performed by determining the absorbance of several concentrations of glucose standards at 483 nm (0, 20, 40, 80, 120, 160, 200 μg ml^-1^).

## Authors' contributions

LGD conceived the experimental design, set up mRNA extraction, performed microarray experiments, RT-PCR, GC-MS, and starch analyses, prepared figures and tables and wrote the initial manuscript draft. JG performed database analysis. MDW and GRC acquired physiological data. RLT performed the identification of housekeeping genes. DRQ supervised the GC-MS analysis. CO performed microarray analyses. DAS contributed to metabolic profiling studies and edited the manuscript. KAS performed all statistical data analysis and edited the manuscript. JCC and GRC contributed equally to the preparation and finalization of the manuscript and conceived the study. All authors have read and approved the final version of the manuscript.

## Supplementary Material

Additional file 1 **Quality control of Vitis GeneChip^® ^genome arrays**. The data provided represent the quality controls and commercial specifications of the 21 arrays used in this study. Slide 1. A) Box plot of raw PM (perfect match) probe intensities before and after RMA normalization. Each color indicates a set of three biological replicates. B) RNA degradation plot for all 21 arrays. All lines have similar shapes and similar variation between highest and lowest points. C) Commercial specifications of the Affymetrix *Vitis *GeneChip^® ^version 1.0. Click here for file

Additional file 2 **Extensive list of transcripts differentially expressed along berry development**. The data provided represent the lists of transcripts that fulfilled the ANOVA filter. Table 1: List of probe sets that passed the ANOVA filter. Table 2: List of Unigenes that passed the ANOVA filter. Table 3: List of probe sets that passed the two-fold ratio (TFR) or greater filter for transcript abundance changes between two stages over berry development. Table 4: List of Unigenes according to Profile number that passed the two-fold ratio (TFR) or greater filter for transcript abundance changes between two stages over berry development. Click here for file

Additional file 3 **Principal component analysis of transcriptomic behavior during grape berry development**. Hybridization data from each biological replicate were projected as two graphs according to the A) first and second and B) second and third principal components arranged in descending order of variance. These first three principal components allowed clear distinction of the seven developmental stages with spots representing data from each biological replicate: E-L stage 31 (light green), 32 (dark green), 33 (brown), 34 (burgundy), 35 (yellow), 36 (light purple), and 38 (orange). Analysis was performed using GeneANOVA software [118]. Click here for file

Additional file 4 **Template profiles used for PTM analysis**. The data provided represent the schematic trends of transcript profiles across berry development used for defining the template profiles. Phases are indicated as I, II, or III. Numbers indicated E-L stages 31 to 38. Pink shading indicates véraison (E-L stages 34 to 35). Click here for file

Additional file 5 **Supplemental data related to the functional analyses of the Unigenes and to real-time RT-PCR**. The data provided represent supplemental data related to Figures 3 and 10. **Table 1**. Attribution of the 20 profiles to Phase I, II or III according to criteria cited in Methods. **Table 2**. *p*-values of the Chi-squared tests of distribution of Unigenes within the three main phases of berry development (I, II and III) for each functional category. Differences in distribution considered as significant are indicated by orange shading. Only Unigenes clustered into the 20 PTM profiles were used for this analysis. **Table 3**. A list of primers used for quantitative real-time RT-PCR experiments. Click here for file
