# Supplementary material for: Transcriptomic and metabolite analyses of Cabernet Sauvignon grape berry development
Source: BMC Genomics. 2007 Nov 22;8:429. doi: 10.1186/1471-2164-8-429 (PMC2220006; doi:10.1186/1471-2164-8-429)
Supplement: Additional file 1 — Quality control of Vitis GeneChip® genome arrays. The data provided represent the quality controls and commercial specifications of the 21 arrays used in this study. Slide 1. A) Box plot of raw PM (perfect match) probe intensities before and after RMA normalization. Each color indicates a set of three biological replicates. B) RNA degradation plot for all 21 arrays. All lines have similar shapes and similar variation between highest and lowest points. C) Commercial specifications of the Affymetrix Vitis GeneChip® version 1.0. [file 1471-2164-8-429-S1.ppt]

## Slide 1
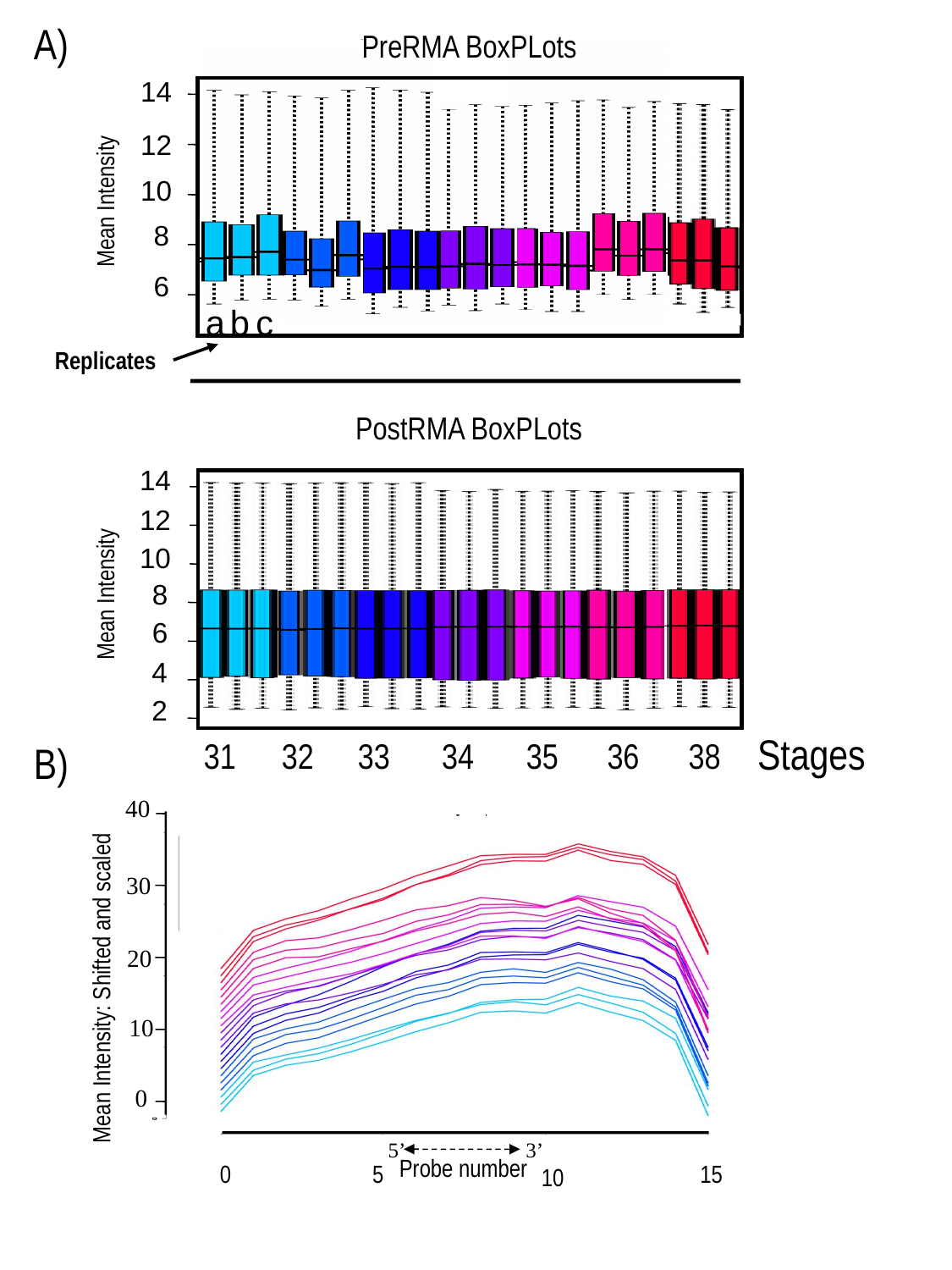

A)
PreRMA BoxPLots
14
12
10
Mean Intensity
8
6
a
b
c
Replicates
PostRMA BoxPLots
14
12
10
8
Mean Intensity
6
4
2
Stages
31
32
33
34
35
36
38
B)
40
30
20
Mean Intensity: Shifted and scaled
10
0
3’
5’
Probe number
0
5
15
10

## Slide 2
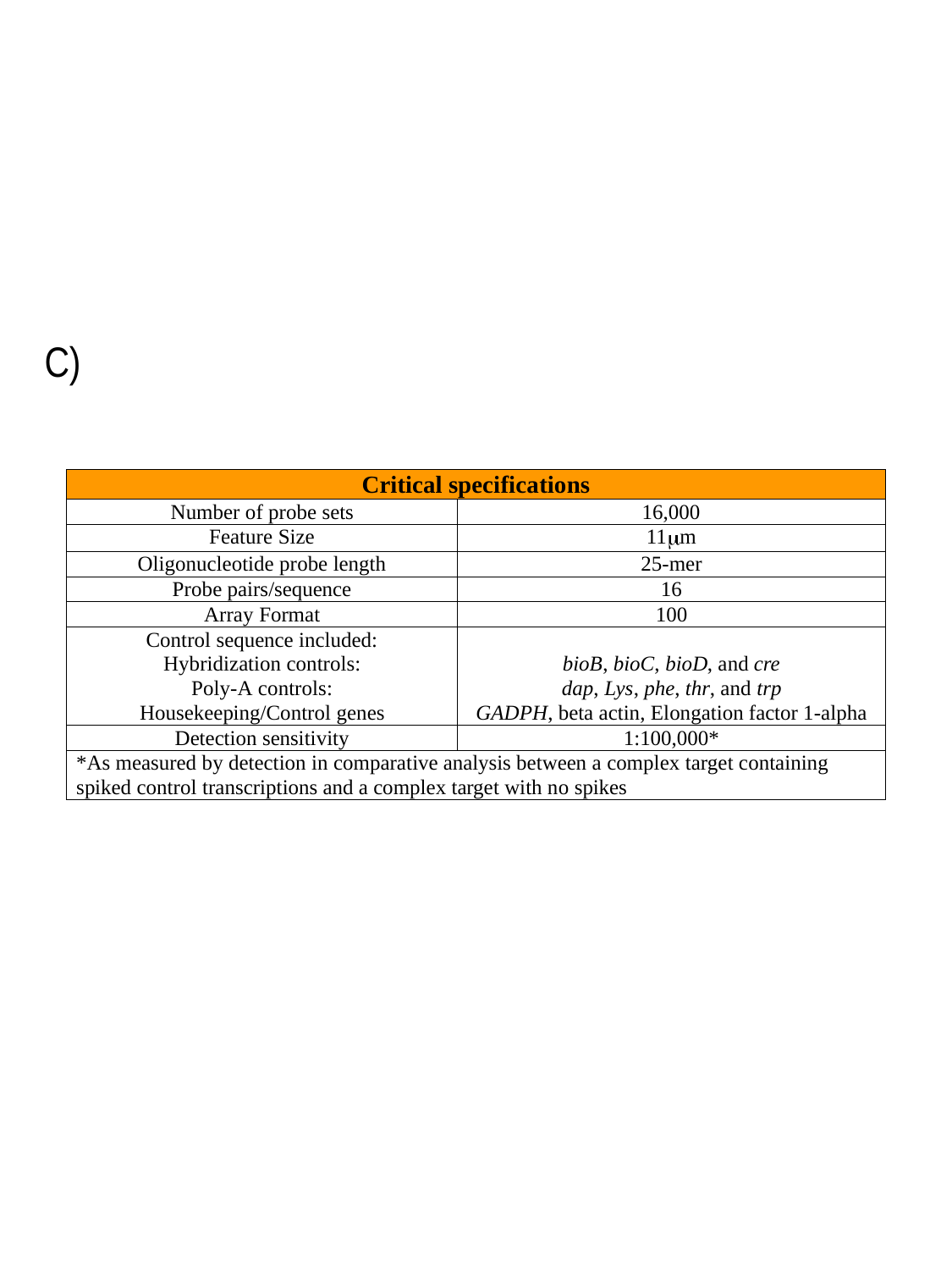

C)
