# Supplementary figures and images for: Transcriptomic and metabolite analyses of Cabernet Sauvignon grape berry development
Source: BMC Genomics. 2007 Nov 22;8:429. doi: 10.1186/1471-2164-8-429 (PMC2220006; doi:10.1186/1471-2164-8-429)

## Slide 1
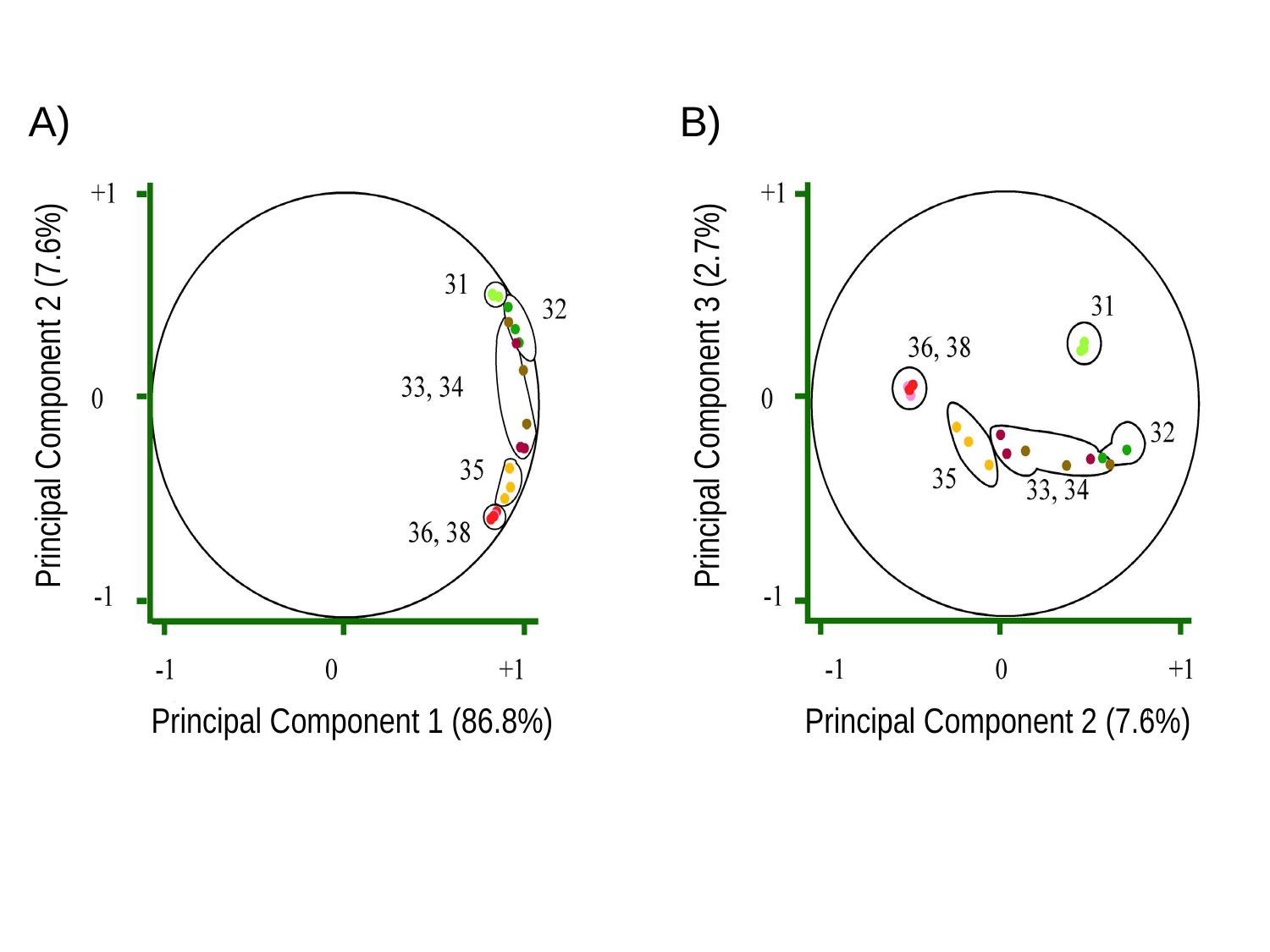

A)
B)
Principal Component 2 (7.6%)
Principal Component 3 (2.7%)
Principal Component 1 (86.8%)
Principal Component 2 (7.6%)

Supplement: Additional file 3 — Principal component analysis of transcriptomic behavior during grape berry development. Hybridization data from each biological replicate were projected as two graphs according to the A) first and second and B) second and third principal components arranged in descending order of variance. These first three principal components allowed clear distinction of the seven developmental stages with spots representing data from each biological replicate: E-L stage 31 (light green), 32 (dark green), 33 (brown), 34 (burgundy), 35 (yellow), 36 (light purple), and 38 (orange). Analysis was performed using GeneANOVA software [118]. [file 1471-2164-8-429-S3.ppt]

## Slide 1
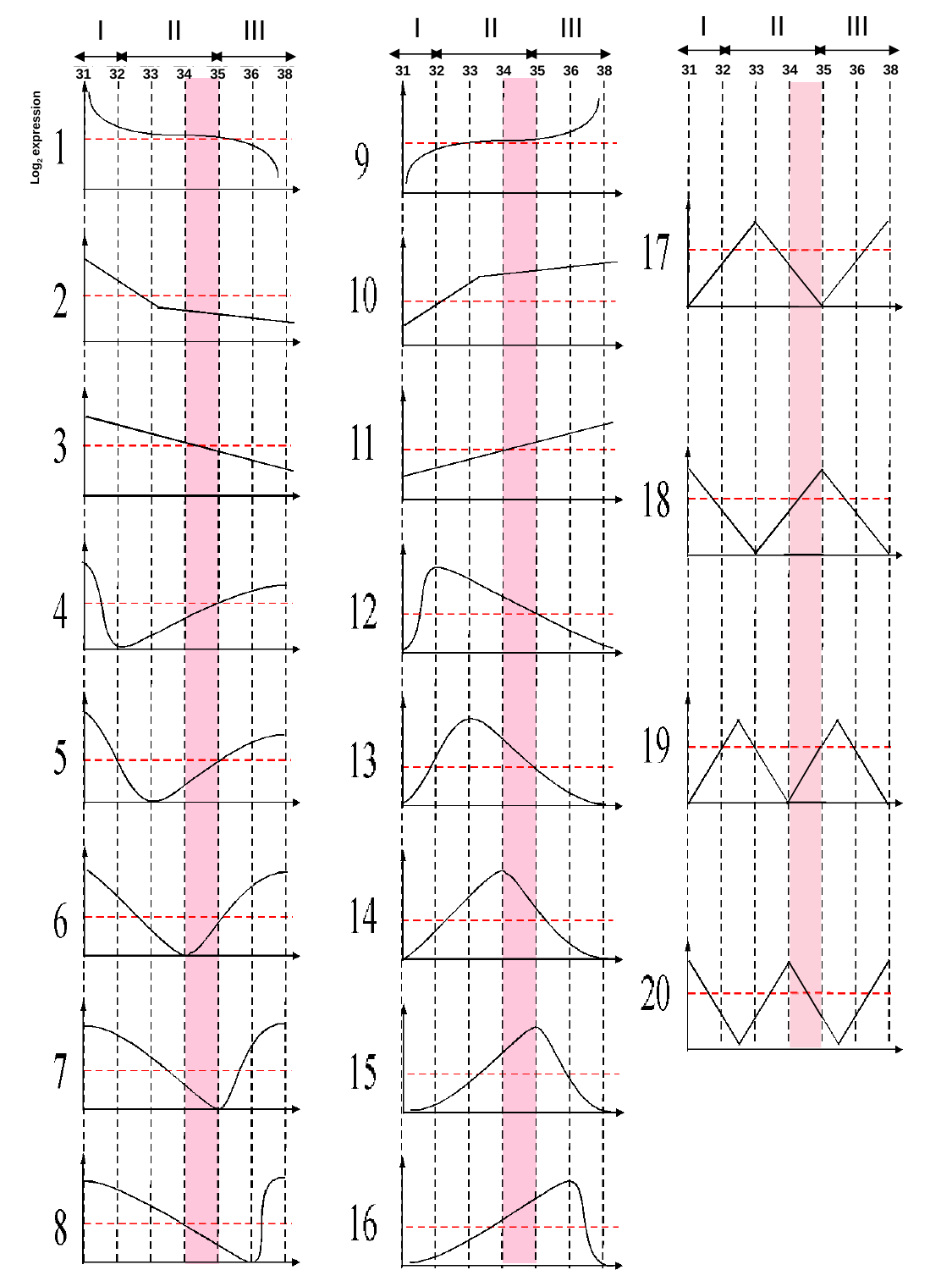

I
II
III
I
II
III
I
II
III
31
32
33
34
35
36
38
31
32
33
34
35
36
38
31
32
33
34
35
36
38
Log2 expression

Supplement: Additional file 4 — Template profiles used for PTM analysis. The data provided represent the schematic trends of transcript profiles across berry development used for defining the template profiles. Phases are indicated as I, II, or III. Numbers indicated E-L stages 31 to 38. Pink shading indicates véraison (E-L stages 34 to 35). [file 1471-2164-8-429-S4.ppt]
