# Supplementary material for: Transcriptomic and metabolite analyses of Cabernet Sauvignon grape berry development
Source: BMC Genomics. 2007 Nov 22;8:429. doi: 10.1186/1471-2164-8-429 (PMC2220006; doi:10.1186/1471-2164-8-429)
Supplement: Additional file 5 — Supplemental data related to the functional analyses of the Unigenes and to real-time RT-PCR. The data provided represent supplemental data related to Figures 3 and 10. Table 1. Attribution of the 20 profiles to Phase I, II or III according to criteria cited in Methods. Table 2. p-values of the Chi-squared tests of distribution of Unigenes within the three main phases of berry development (I, II and III) for each functional category. Differences in distribution considered as significant are indicated by orange shading. Only Unigenes clustered into the 20 PTM profiles were used for this analysis. Table 3. A list of primers used for quantitative real-time RT-PCR experiments. [file 1471-2164-8-429-S5.doc]

**Table 1**:

| Profiles | Phase I | Phase II | Phase III |
| --- | --- | --- | --- |
| **1** | O | - | - |
| **2** | O | - | - |
| **3** | O | - | - |
| **4** | - | - | O |
| **5** | - | - | O |
| **6** | O | - | - |
| **7** | O | - | - |
| **8** | O | - | - |
| **9** | - | - | O |
| **10** | - | - | O |
| **11** | - | - | O |
| **12** | - | O | - |
| **13** | - | O | - |
| **14** | - | O | - |
| **15** | - | - | O |
| **16** | - | - | O |
| **17** | - | O | - |
| **18** | - | - | O |
| **19** | - | - | O |
| **20** | O | - | - |

**Table 2**:

| Functional Category | (Phase I-II) | (Phase I-III) | (Phase II-III) |
| --- | --- | --- | --- |
| Metabolism (1) | 0.5301 | 0.0034 | 0.0207 |
| Energy (2) | 0.6904 | 0.02 | 0.3768 |
| Storage protein (4) | 0.0001 | 0.2701 | 0.004 |
| Cell Cycle and DNA processing (10) | 0.0355 | 0.4522 | 0.018 |
| Transcription (11) | 0.0274 | 0.0036 | 0.613 |
| Protein Synthesis (12) | 0.2617 | 0.0045 | 0.6411 |
| Protein Fate (14) | 0.1432 | 0.0001 | 0.3965 |
| Protein With Binding Function (16) | 0.7491 | 0.0697 | 0.5142 |
| Protein Activity Regulation (18) | 0.6537 | 0.9019 | 0.6993 |
| Transport Facility (20) | 0.8449 | 0.0449 | 0.362 |
| Signal Transduction (30) | 0.2881 | 0.8993 | 0.3174 |
| Cell Rescue (32) | 0.8091 | 0.6977 | 0.6481 |
| Interaction With The Cellular Environment (34) | 0.7923 | 0.2576 | 0.7292 |
| Interaction With The Environment (36) | 0.6836 | 0.9488 | 0.708 |
| Transposable Elements (38) | 0.2277 | 0.001 | 0.4903 |
| Cell Fate (40) | 0.4982 | 0.3962 | 0.8128 |
| Development (41) | 0.9631 | 0.5576 | 0.7851 |
| Biology of Cellular Components (42) | 0.0111 | 0.0001 | 0.7444 |
| Cell Type Differentiation (43) | 0.5715 | 0.1687 | 0.8757 |

Table 3:

| **Unigene (VvGi5)** | **Name** | **Probesets** | **Forward** | **Reverse** |
| --- | --- | --- | --- | --- |
| TC51695 | Flavonoid 3',5'-Hydroxylase | 1607760_at | 5'-CCACACCACTGAACCTTCCT-3' | 5'-TGCCCATATGTTCACGCTAA-3' |
| TC54034 | Cinnamoyl Alcohol Dehydrogenase | 1616880_at | 5'-GCTTGGAGATTTAGGCGTTT-3' | 5'-AATAGGAGCTTTGGCCCATT-3' |
| TC62182 | Nitrate/ Chloride transporter | 1613896_at | 5'-ACTACTTGGTTGCTGCTTTGG-3' | 5'-TTCTCCAGGCTCACCTCAAG-3' |
| TC57228 | AtWRKY7 | 1611650_at | 5'-AGAAGCCGCTGGTCTCATC-3' | 5'-ACAGGGAGAGCATGGAGTTG-3' |
| TC55943 | Serine/threonine kinase | 1611342_at | 5'-CTGTTGAATGGGAGGCAACT-3' | 5'-GCTGAAGCAGTGGGATTGAT-3' |
| TC51776 | Aquaporin PIP1.1 | 1615722_s_at | 5'-ATCTTCTGGGTGGGACCATT-3' | 5'-CCAGCACCACTCATCATCAC-3' |
| TC61058 | MYB | 1614931_at | 5'-TAGGCAAGGGCAAGAACAGT-3' | 5'-GGGCAATACCACTTCATACCA-3' |
| TC68311 | Protein Phosphatase 2C | 1612132_s_at | 5'-TTGTCCGGTTCAAGGGTTAG-3' | 5'-GGCCAGGTATTGGTGACAGT-3' |
| TC63764 | Ferulate-5-hydroxylase | 1614502_at | 5'-CGACGTGTTCGGACTCACT-3' | 5'-CCAAACAGTCCCTCCATGAT-3' |
| TC58595 | Type B Response Regulator (ARR5) | 1608140_at | 5'-GTCACCAGCACCATCACTTG-3' | 5'-CCCACAATCTAGGTCGCTTT-3' |
| TC63891 | Osmotin precursor | 1606794_at | 5'-TGCCCTGATGCTTACAGCTA-3' | 5'-AGAGGCTTTATGGGCAGAAGA-3' |
| TC53526 | Sucrose Synthase | 1616700_at | 5'-GTCTACGGGTTCTGGAAGCA-3' | 5'-GCAGACACGTACAGCTCCTTT-3' |
